# The macroeconomic determinants of trade openness in Latin American countries: A panel data analysis

**DOI:** 10.12688/f1000research.153690.2

**Published:** 2024-10-21

**Authors:** Rogger Orlando Morán Santamaría, Yefferson Llonto Caicedo, Francisco Eduardo Cúneo Fernández, Lizana Guevara Nikolays Pedro, Castro Mejía Percy Junior, Milagros Judith Pérez Pérez, Lindon Vela Meléndez, Moises Elias Montenegro López

**Affiliations:** 1 La Libertad, Universidad Cesar Vallejo, Trujillo, La Libertad, Peru; 2 Lambayeque, Universidad Nacional Pedro Ruiz Gallo, Lambayeque, Lambayeque, Peru

**Keywords:** Trade openness; impact; panel data

## Abstract

**Background:**

Trade openness shows a positive impact on economic growth, supported by economic theory, and export diversification and economic complexity show a positive dynamic in trade openness in the world; however, a specificity is generated in South American countries. Therefore, the objective of the research is to analyse the macroeconomic determinants of trade openness in Latin American countries.

**Methods:**

The research approach was quantitative and explanatory using panel data methodology from the databases of the World Bank, Harvard University and the Economic Commission for Latin America and the Caribbean for the period 2000-2020.

**Results:**

The fixed effects panel data model showed that the variables that had a negative impact on trade openness were GDP, the economic complexity index and the logistic performance index, while the variables that had a positive impact were exports of high-tech products (a proxy for innovation), exports, imports, research and development expenditure and interregional trade in goods.

**Conclusions:**

Therefore, during the analysis period of 2000-2020 in South America, based on the panel data analysis under fixed effects, a total of 8 countries had a negative impact on trade openness, and only the economies of Chile, French Guiana, and Brazil had a positive impact on trade openness; these economies are characterized by their better performance in the economic complexity index, their higher percentage of budget for research and development expenses, and their trade policies oriented towards the industrialization of their value-added products.

## 1. Introduction

Trade openness in countries around the world has shown significant relevance in addressing one of the greatest global crises, where the acute and real problem of global food security, coupled with rising inflation, demonstrates that trade policies not only ensure adequate access to food but also compel a consideration of the link with economic complexity, which in turn requires research and development and the creation of an innovation climate that impacts economic growth ( Bardi & Hfaiedh, 2021; Fusco et al., 2020; Gallego & Zofío, 2018; Tabash et al., 2022). Moreover, sustainable trade practices are crucial for promoting long-term economic resilience, environmental protection, and social well-being, aligning with the broader goals of sustainable development.

The debate focuses on the so-called resource curse, where Latin American economies show that after the decades of the eighties and nineties, they developed a strategy for diversifying exports and boosting the reorientation of the external sector where goods of medium and high technological intensity are produced; while other countries such as Peru, Ecuador, Bolivia, Chile, Colombia, Paraguay, and Venezuela based their trade basket on the primary sector as the support of the export industry ( Alvarado & Guzmán, 2019; Gnangnon, 2022; Tabash et al., 2022).

The growing international trade flow, the development of technological capabilities, research and development, and economic complexity have had a direct and significant impact on sustainable economic growth. From the theoretical perspective of economic policy for sustainable growth, trade policy plays a fundamental role in the state, as market economies require the existence of an efficient and effective state that can ensure compliance with free trade rules ( Cetin et al., 2018; Chávez, 2021; Lima & Alvarez, 2018).

In the region of South America, according to data from the Economic Commission for Latin America (2021) in Argentina, the average growth of trade openness, which allows assessing the magnitude of foreign trade relative to a country’s internal production, was 28% in the period 1990-2022, being volatile and reaching the highest percentages of openness degree in the period 2002-2008 then showing a decline and reaching 31.7% in 2022.

In the case of Bolivia, the average trade openness indicator reached 60.1% in the period 1990-2022, and from 1990 to 2008 showed an increasing trend, reaching 67.8% in 2022. For Brazil, the average growth of trade openness was 24.3% in the period 1990-2022, with an increasing trend until reaching 38.8% in 2022.

Meanwhile, in Chile, during the period 1990-2022, an average of 62.4% was reached, achieving 75% in 2022; in the case of Colombia, the average reached during the mentioned period is 35.3% and in 2022 reached 48.4%. In the case of Ecuador, the average trade openness indicator reaches 50.9% and in 2022 reached 57.7%; for Paraguay, it reached a degree of openness of 82.8% in the period 1990-2022; while Peru reached 42.4% in the same period; and Uruguay reached 52.7%.

The arguments in favor of trade liberalization start from the greater accumulation of physical capital but face diminishing returns; while the growth lever bets on the accumulation of knowledge, which generates significant and dynamic impacts that lead companies needing access to large markets to innovate and become more competitive given the fluidity towards the ability to learn and assimilate new technologies ( Corrales Sillo, 2021; Mesquita & Stein, 2019).

While trade openness has empirically shown positive externalities for economies, such empirical evidence still lacks clear empirical factors, especially in Latin American economies where the impact is estimated to be an acceleration of between 0.6 and 0.7 percentage points on average to the annual growth of per capita gross domestic product. This is mainly due to the role of political, institutional, and structural factors related to technology and innovation; given the particularities of the countries that make up the South American region, the low levels of human capital and the distant technological frontier may have affected trade openness, diminishing the positive impact of trade liberalization as addressed by economic theory ( Grossman & Helpman, 2015; Goldberg & Pavcnik, 2016).

However, there is no doubt that trade openness has led to leaving behind apocalyptic decades in Latin America; it was the shift in trade policy that originated a positive contribution to development and well-being ( Herreros and Rosales, 2013; Lopez & Munoz, 2020); as Boloña (1993) held, “precisely because we are a small country in economic terms, we must diversify our markets, expand the scale of our economy, benefit from international markets to grow and accumulate capital, to overcome poverty and achieve the desired levels of well-being and prosperity“(p.13).

Falcón (2021) begins from the challenge of directing trade policy towards a greater degree of diversification of productive matrices that allows countries to achieve greater economic complexity based on a greater accumulation of knowledge and not just on the aspect of more physical capital or working capital, leading to bet on technological development and the transformation of the basket of exportable products leading to manufactured goods with added value, surpassing the threshold of commodities and developing comparative advantages, with a relationship between trade openness, economic complexity, innovation, research and development, and economic growth ( Lee & Lee, 2020; Shahzad et al., 2022); understanding the particularity of each country in its productive transformation based on the existing connection between the variables under study, which will lead to a public policy decision to achieve the transformation of the external sector for sustainable growth with development prospects in emerging economies ( Yang et al., 2023; Cheong, 2023).

In this context, the present article aims to analyze the macroeconomic determinants of trade openness in Latin American countries; thus, posing the question: What are the macroeconomic determinants of trade openness in Latin American countries: a panel data analysis? considering the explanatory variables of economic complexity, innovation, research and development, and economic growth for the mentioned period.

The contribution, considering the specificity of the model that proves to be crucial for policymakers, takes into account that the study is only conducted in South American countries over a specific period from 2000 to 2020 and contributes to the empirical evidence of considering the importance of better integrating into the global economy by achieving greater competitiveness and considering trade openness from an important position in income distribution and poverty reduction that benefits generating investment and dynamism in international trade in the region.

## 2. Literature review

Recent empirical evidence has been highlighting the importance of the external sector through trade openness as an economic policy in the growth of emerging economies like those in Latin America, in a current scenario of health crisis and food security, as well as economic uncertainty that has underscored the importance of diversification and complexity of exports, leading to a restructuring of the productive matrix which in turn requires the accumulation of physical capital and the expansion of capabilities. This necessitates developing a climate of business innovation as well as allocating more resources to research and development ( Carrasco, 2021; Chávez, 2021; Bashir et al., 2020; Chacha et al., 2024; Guo et al., 2023; Mania & Rieber, 2019; Nieminen, 2020).

In the international environment, Wang et al. (2023) conducted a study evaluating economic complexity, trade diversification, renewable energy consumption, and environment-related taxes to generate growth during the period 1995-2018. It was identified that the aforementioned variables significantly propelled the growth of BRICTS countries, generating important policy implications for achieving marginal contributions.

For Li and Rigby (2023) economic growth has a positive and significant relationship with complex technological diversification. Evidence of spatial autocorrelation and endogeneity confirms the positive link between technological advancement and economic growth in China between the periods 1991-1995 and 2011-2015, where measuring knowledge complexity has generated a positive relationship, the result of the regression showing that intelligent specialization has led to a knowledge development trajectory for achieving regional competitive advantage.

The nexus between innovation, trade openness, financial development, and economic growth using the panel VAR approach in 11 European countries over the period 2001-2016 reveals a unidirectional relationship between economic growth and financial development; as well as trade with economic growth and the negative relationship between innovation and economic growth and between trade and economic growth. Thus, the important role of each country depends on the role that innovation and productivity play, recommending that the quality of institutions is fundamental to improving the regulatory environment to achieve optimal trade openness and generating incentives for innovation in local companies to address the negative effects generated by trade openness ( Mtar & Belazreg, 2023).

Various studies show the relationship between the explanatory variables of economic complexity, innovation, research and development, and economic growth.

In 28 African countries, considering a panel data analysis, the mediating effect of economic complexity and the natural resource-based growth model for the period 1995-2019 has been considered. The empirical evidence indicates that the positive effect of natural resources on economic growth in the short and long term, generating robust results, where economic complexity seeks to formulate policies to achieve links between the variables of natural resources and economic growth using economic complexity ( Mesagan & Vo, 2023).

In Russia, high-tech companies that generate new products and markets are a key factor in long-term economic development, where the region’s export basket reflects operations in the directions of technological development to diversify a technological chain with higher added value and hence competitiveness of export-oriented products, creating knowledge-intensive and high-technology sectors aimed at developing local advantages and market expansion with interregional cooperation ( Goryushkin & Khalimova, 2023).

Thus, the diversification of exports and economic complexity is receiving relevance in the scientific literature so that the results of structural equation models generate a positive relationship between economic complexity and per capita economic growth of each country. The results show that both the level of per capita gross domestic product and economic vulnerability infer on economic complexity, given the economic challenges faced by countries with low growth, high unemployment, rising debt, and regional vulnerability ( Ben Saad et al., 2023).

For Rahman et al. (2023) in Bangladesh, economic growth is argued in terms of technological innovation, foreign direct investment, trade, and human capital over the period 1990-2020, using time series for the dynamic simulation ARDL method, where cointegration and the symmetrical long- and short-term associations between the variables, consider that technological innovation and human capital have a long-term relationship with economic development in the country. Generating the contribution of a significant correlation between economic growth and the described variables in the short term, considering the robustness of the study.

For Bardi and Hfaiedh (2021) evidence shows a positive impact of trade openness on economic growth, revealing unidirectional causality based on economic theory where trade openness has positive effects on economic growth ( Barro & Sala, 2009; Lucas, 1988; Romer, 1986; Banday et al., 2021; Zaman et al., 2021).

Canh and Thanh (2022) for their part, evidence that the diversification of exports and economic complexity show a positive dynamic, presenting bidirectional causality given the role played by the quality of export products which is measured from the productive capacities of a country to add value to its export products achieving more competitiveness through the structural transformation of economies and the diversification of the productive matrix of countries ( Armas et al., 2020; Shahzad et al., 2022; Lapatinas et al., 2019; Koch, 2021).

For Mesquita and Stein (2019) they consider that although the great trade liberalization in Latin America has led to a positive impact on economic growth and well-being being the channel that helped recover much of the economies of Latin America from their participation in trade in the gross domestic product in the region that reached 28% they still face challenges in terms of technological aspect and innovation to continue the solid growth of international trade; this because there are comparative advantages that allow productivity leaps ( Llanos & Brown, 2021; Vela et al., 2021).

For Chávez (2021) considers in the panel data model two related equations innovation as the dependent variable and trade openness as the independent variable and as a second equation was obtained as the dependent variable to the predictions of innovation and trade openness as the explanatory variable.

Meanwhile, Fusco et al. (2020) considers in their analysis of dynamic panel data where they analyze food security and trade openness developing three regression equations with indicators of trade openness, tariffs, and globalization. While Cetin et al. (2018) considers economic growth, energy consumption, trade openness, and financial development as explanatory variables and carbon emissions as the dependent variable for the case of Turkey.

## 3. Theoretical basis

### 3.1 Trade openness

Gräbner et al. (2021) believe that international trade generates economic growth, suggesting that while trade openness is not a direct or robust determinant in economic growth, it contributes significantly to it. By leveraging growth potential, greater trade openness generates ideas and knowledge, along with their respective technical advancements, to open up new goods and services in international trade, contributing from the theory of the openness paradigm and initiating a debate on the causality of growth ( Silajdzic et al., 2018).

### 3.2 Economic growth

Hartmann (2014) from the contribution of economic theory, considers that growth is linked to the increase in production, income, and consumption. Meanwhile, the classic view of Smith (1976) considers that both the division of labor and trade are key aspects to achieving growth, explained by the classical factors of capital, labor, and land, but with an additional factor related to technology ( Solow, 1956). Subsequently, approaches to economic growth explained by technological progress emerged, providing a different perspective on long-term growth ( Romer, 1986) with technology as an exogenous factor of growth rather than an endogenous one.

### Economic complexity

Hausmann et al. (2020) consider the theory of economic complexity, which involves moving away from traditional factors to focus on knowledge as the main value generator for achieving a new structural transformation that allows diversifying the productive matrix and empowering the aspect of greater competitiveness. For Hidalgo and Hausmann (2009) economic complexity refers to the set of a region’s export products that shows a country’s productive capacity based on existing capabilities and know-how, implying that the more diverse and value-added the export products, the more productive the region is considered.

### Innovation

The paradigm of innovation and structural changes have become prominent in recent literature due to the composition of new economic systems where creation, innovation, and entrepreneurship have led to major changes in countries like India, China, and the United States, whose knowledge-based growth has led to their exponential development. Schumpeter (1941) in his theory of economic development addressed that innovation, as well as inventions by scientists, would lead to creating entirely new investment, growth, and employment opportunities. According to Schumpeter and Swedberg (2003) the innovation process can be divided into four dimensions: invention, innovation, diffusion, and imitation.

### Research and development

Elfaki and Ahmed (2024) consider that as a result of technological adoption, these elements are fundamental for the economic growth of any nation. In a world where global competition is increasingly intense, the ability to innovate and continuously improve becomes a determining factor for economic success. R&D drives progress, not only through the creation of new products and services but also by optimizing existing processes, thus increasing productivity and efficiency across various industries.

Companies that allocate resources to research and development tend to be at the forefront of creating novel products and services that better meet consumer needs. This not only makes them more competitive but also enables the creation of new industries and markets, which in turn can lead to sustained economic growth.

Being the hypothesis that economic complexity, innovation, research and development, and economic growth are the determinants of trade openness.

## 4. Methods

### 4.1 Methodological design

*4.1.1 Type of research*

A quantitative approach is considered, with a type of explanatory research that corresponds to identifying the impact of economic complexity, innovation, research and development, and economic growth on the trade openness of South American countries during the period 2000-2020.

*4.1.2 Research design*

The variables analyzed have not been modified, and the research is a non-experimental design; therefore, it has been verified that it was conducted in a specific context of the South American countries and the interaction of the unmanipulated variables ( Hernández et al., 2018).

*4.1.3 Population*

The population involves a total of 2079 data points across 9 analyzed variables considering a total of 11 South American countries, noting that we have 231 data points per variable for each country under analysis during the 2000-2020 period.

*4.1.4 Sample*

The sample is equal to the population and comprises a total of 2079 data points across 9 analyzed variables considering a total of 11 South American countries, noting that we have 231 data points per variable for each country under analysis during the 2000-2020 period.

*4.1.5 Unit of analysis*

The unit of analysis consists of each South American country, involving a total of 11 countries analyzed during the 2000-2020 period.

*4.1.6 Data collection techniques and instruments*

For measuring the variables, documentary analysis was used as the technique, and the observation guide served as the instrument, which is a tool used to collect data systematically and structured during an observation or study.

*4.1.7 Data collection procedures*

The data obtained from the World Bank corresponded to the explanatory variables of gross domestic product, imports, exports, high-tech product exports, research and development expenditure, and the logistics performance index; as well as from the Harvard University Economic Complexity Atlas, where information on the economic complexity index was obtained. Regarding the interregional trade of goods variable, it was obtained from the database of the Economic Commission for Latin America and the Caribbean during the mentioned analysis period.

A panel data model is used, where from this model, the impact of economic complexity, innovation, research and development, and economic growth on the trade openness of the South American countries during the 2000-2020 period is quantified. To perform the estimates consistently and achieve a proper analysis, the variables have been transformed into logarithms such as gross domestic product, imports, exports, high-tech product exports, and total interregional trade of goods.

### 4.2 Data analysis

The databases used for this research stem from international institutions such as the World Bank, Harvard University, and the Economic Commission for Latin America for the period 2000-2020.

In the estimation, we have used the panel data model based on economic literature that demonstrates a positive impact of trade openness on economic growth ( Romer, 1986; Barro & Sala, 2009; Lucas, 1988; Katz, 1999).

Highlighting the importance of export diversification and complexity, leading to restructuring the production matrix, which in turn requires the accumulation of phys-ical capital and expansion of capacities, thereby necessitating the development of a climate of entrepreneurial innovation, as well as allocating greater resources to research and development ( Carrasco, 2021; Chávez, 2021).

Equation 1 establishes the relationship of the dependent variable trade openness with exports, imports, and gross domestic product per country in South America i =1, 2, …………. 11 in the year t = 2000-2020.

ACi,t=Bo+B1(Exporti,t)+B2(Importi,t)+B3(Pbii,t)+ei,t (1)

On the other hand, the incorporation of the variable’s economic complexity (eco-nomic complexity index), innovation (proxy: exports of high-tech products), research and development (percentage expenditure on research and development), the logistic performance index, and total interregional trade of goods are evidenced in the following equation:

ACi,t=Bo+B1(Exporti,t)+B2(Importi,t)+B3(Pbii,t)+B4(ICEi,t)+B5(Export_teci,t)+B6(i_di,t)+B7(i_logi,t)+B8(c_interi,t)+ei,t (2)

Next, the Generalized Least Squares (GLS) model is estimated because the equations present problems of heteroskedasticity and autocorrelation detected with the Breusch and Pagan (1979) (as cited in Onali et al., 2017) and Wooldridge (2010) Lagrange Multiplier tests. Furthermore, to choose between a fixed or random effects model, the Hausman test (1978) (as cited in Onali et al., 2017) is utilized.

The variables used in the proposed panel data model are detailed in the following Table 1.

**Table 1.** Variables used in the model.

Variable	Description	Source
Trade openness (ac i,t)	Economic openness index measuring the degree of a country's economic openness; calculated as the sum of exports plus imports divided by gross domestic product.	World Bank 2000-2020
Exports (Export i,t)	Merchandise exports (US$ at current prices)	World Bank 2000-2020
Imports (Import i,t)	Merchandise imports (US$ at current prices)	World Bank 2000-2020
Gross Domestic Product (Pbi i,t)	Value of all goods and other market services exported to the rest of the world by department, US$ at current prices	World Bank 2000-2020
Export of High-Tech Products (Export_tec i,t)	Value of exports of high-tech products, which are highly research and development intensive products	World Bank 2000-2020
Research and Development Expenditure (i_d i,t)	Expenditure on research and development (% of GDP)	World Bank 2000-2020
Logistic Performance Index (i_log i,t)	Logistic Performance Index: Total (From 1=low to 5=high)	World Bank 2000-2020
Economic Complexity Index (ice i,t)	Economic Complexity Index, a set of export products from a region showing a country's productive capacity based on existing capabilities and know-how	Harvard University, 2000-2020
Total Interregional Trade of Goods (c_inter _i,t_)	Value of trade in goods between countries, FOB exports in millions of dollars	Economic Commission for Latin America, 2000-2020

Note: Own elaboration with data from World Bank, Harvard University, Economic Commission for Latin America, 2000-2020, using Eviews 12.

**Figure 1.**
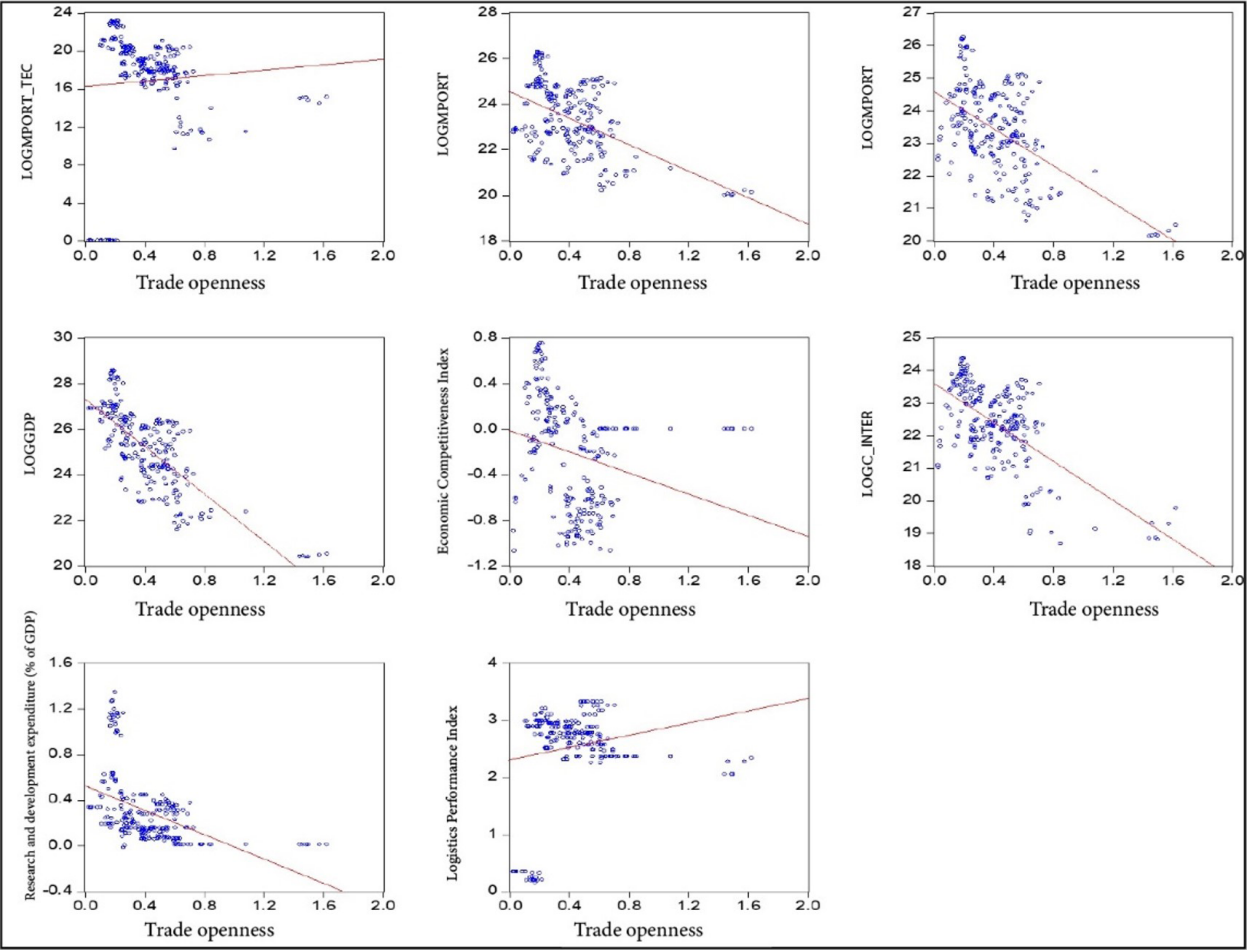
Correlation of model variables. Note: Own elaboration with data from World Bank (2024), Harvard University (2024), Economic Commission for Latin America (2024), for the period 2000-2020, using Eviews 12.

The data were analyzed using an econometric panel data model that captures the bidimensionality of the data. This model allows for the quantification of the impact of economic complexity, innovation, research and development, and economic growth on the trade openness of South American countries during the period 2000-2020. The data were obtained from the World Bank, Harvard University, and the Economic Commission for Latin America and the Caribbean and processed in Eviews 12 and is used for the estimation of the panel data model, considering the control variables that are subjected to the proposed equations to analyze the interrelationship between the variables ( Figure 1). This allows for both descriptive statistical treatment and the estimation of the proposed panel data model and the Generalized Least Squares (GLS) model. An academic license for the use of Eviews 12 software is available, registered under the name Lindon Vela Meléndez. The license details are as follows: Serial number: Q1208886 - D49010AF - 9D854485. The software can be downloaded from the following link: http://www.eviews.com/download/student12.

Furthermore, the Generalized Least Squares (GLS) model was employed due to issues of heteroscedasticity and autocorrelation identified in the structural equations. These issues were detected using the Breusch and Pagan (1979) (as cited in Onali et al., 2017) Lagrange Multiplier test and the Wooldridge (2010) test. Additionally, the Hausman (1978) (as cited in Onali et al., 2017) test was used to decide between a fixed or random effects model, which helps in choosing the most suitable model based on the consistency of the estimators.

### 4.3 Ethical considerations

The data for this study were obtained from international institutions such as the World Bank, Harvard University and the Economic Commission for Latin America for the period 2000-2020. Access to the data is public and free of charge on the detailed websites and adheres to rigorous ethical standards to protect the confidentiality of the published data.

In addition, the data from the World Bank, Harvard University and the Economic Commission for Latin America is an effort by international institutions to generate databases that allow interaction with public policy makers.

Although we cannot provide specific details on the process of obtaining the data, it is imperative to indicate that, as prestigious governmental institutions such as the World Bank, Harvard University and the Economic Commission for Latin America, there is a use and policies to access under ethical practices in the generation of data in the respective public repositories and free access and obtaining informed consent.

For the ethical use of public data obtained by the World Bank, Harvard University and the Economic Commission for Latin America, we adhere to the principles of intellectual honesty, truthfulness, transparency, human integrity, respect for intellectual property, justice and responsibility, the study aims to comply with the “Code of Ethics in Research of the Universidad César Vallejo, version 01; by Resolution of the University Council N° 0340-2021-UCV”, code of ethics that was used throughout the development of the scientific article, since as main author we work all scientific activity respecting the code of ethics of the UCV university.

We have used the data from the described repositories for research purposes only, as permitted by the conditions of use by the World Bank, Harvard University and the Economic Commission for Latin America. We have not attempted to modify any data, and our analysis does not include any personally identifiable information.

By using publicly available anonymized data and adhering to ethical principles in our research, we aim to minimize any potential ethical issues while leveraging valuable information for the benefit of research in economic science.

## 5. Results

**Figure 2.**
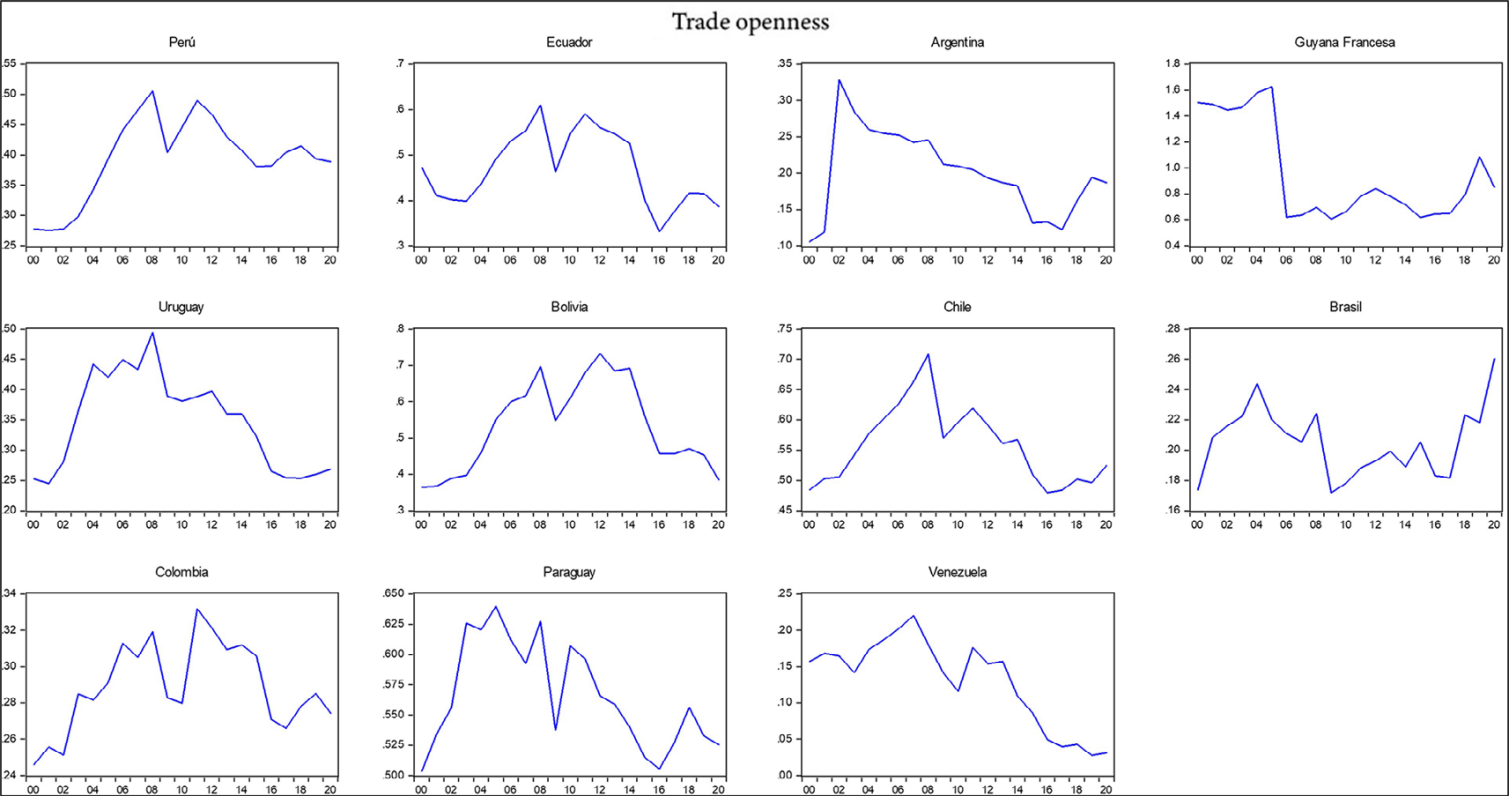
Trade openness index by country. Note: Own elaboration with data from World Bank (2024), Harvard University (2024), Economic Commission for Latin America (2024), for the period 2000-2020, using Eviews 12.

The research utilizes data sourced from the World Bank, Harvard University, and the Economic Commission for Latin America and the Caribbean. Descriptive statistics for each variable analyzed are presented in Table 2, including the mean, standard deviation, minimum, maximum, skewness, and kurtosis. Additionally, the correlation matrix between the variables under analysis is provided. It shows that exports, imports, gross domestic product (GDP), the economic complexity index, total interregional trade of goods, and research and development had a negative relationship with trade openness, whereas the export of high-technology products and the logistics performance index had a positive correlation with trade openness during the period 2000-2020. The negative relationship indicates that South American countries, except for Chile and Brazil, have shown less trade openness in their economies over the last decade with respect to the international market. According to the Economic Commission for Latin America and the Caribbean (2021) economies in South America have demonstrated reduced trade openness due to the slowdown in international trade despite their high dependence on imports and extensive trade relations with major global economic powers in a scenario of economic uncertainty and a political shift to the left, which helps to understand which variables are influencing reduced trade openness ( Figure 2).

**Table 2.** Descriptive statistics of the model variables.

	AC	Export_tec	Export	Import	Pbi	ICE	C_INTER	I_d	I_LOG
Mean	0.42	16.87	23.32	23.38	25.13	-0.21	22.34	0.30	2.54
Median	0.39	18.31	23.23	23.35	25.32	-0.10	22.40	0.20	2.76
Maximum	1.63	23.13	26.27	26.25	28.59	0.75	24.39	1.34	3.32
Minimum	0.03	0.00	19.99	20.15	20.38	-1.07	18.68	-0.02	0.15
Std. Dev.	0.26	5.93	1.50	1.34	1.76	0.48	1.22	0.30	0.77
Skewness	1.99	-1.99	-0.15	-0.22	-0.45	0.01	-0.91	1.79	-2.24
Kurtosis	9.47	6.18	2.37	2.70	3.00	1.99	3.74	5.68	7.14
AC	1.00	0.06	-0.50	-0.54	-0.76	-0.25	-0.63	-0.46	0.18
Export_tec	0.06	1.00	0.55	0.26	0.19	0.25	0.30	0.36	0.95
Export	-0.50	0.55	1.00	0.90	0.90	0.33	0.88	0.71	0.45
Import	-0.54	0.26	0.90	1.00	0.92	0.29	0.90	0.65	0.15
Pbi	-0.76	0.19	0.90	0.92	1.00	0.37	0.91	0.72	0.06
ICE	-0.25	0.25	0.33	0.29	0.37	1.00	0.27	0.58	0.18
C_inter	-0.63	0.30	0.88	0.90	0.91	0.27	1.00	0.61	0.17
I_d	-0.46	0.36	0.71	0.65	0.72	0.58	0.61	1.00	0.22
I_LOG	0.18	0.95	0.45	0.15	0.06	0.18	0.17	0.22	1.00
N	231	231	231	231	231	231	231	231	231

Abbreviations used: “N” = Number of observations, “Std. Dev.” = Standard Deviation, “Minimum” = Minimum, “Maximum” = Maximum.

Note: Own elaboration with data from World Bank, Harvard University, Economic Commission for Latin America, 2000-2020, using Eviews 12.

The standard deviation indicates that there is greater variability among the variables of export of high-technology products, exports, imports, gross domestic product, and total interregional trade of goods, with a total of N data points equivalent to 231 observed data per variable representing the total number of 2079 observations across the 9 variables studied during the period 2000-2020 for the 11 South American countries. This variability and the detailed analysis of the correlations provide insights into the complexities of trade dynamics and economic interactions in the region, underscoring the nuanced impact of various economic factors on trade openness.

The estimated regression results, considering the Wooldridge (2010) test, indicate the presence of autocorrelation across the panels. Additionally, the Breusch and Pagan (1979) (as cited in Onali et al., 2017) Lagrange Multiplier test shows that the estimations had issues with heteroscedasticity; these issues have been corrected using Generalized Least Squares (GLS) to address both heteroscedasticity and autocorrelation.

Table 3 displays the results of the panel data estimation, revealing that public health spending, rural population, unemployment, and gross domestic product are statistically significant at the 5% level considering equation 2.

**Table 3.** Regression: Trade openness with explanatory variables.

Explanatory variable	Levels	Fixed effects	Mobile effects
Constant	1.341 *	1.439 *	2.091 *
Log Export_tec	-0.017	0.053 *	0.003
Log Export	0.243 *	0.301 *	0.379 *
Log Import	0.175 *	0.086 *	0.098 *
Log Pbi	-0.425 *	-0.425 *	-0.461 *
ICE	-0.002	-0.078 **	0.057 *
Log C_INTER	0.016	0.005	-0.027
I_d	0.130	0.141 **	0.083
I_LOG	-0.045	-0.127 *	-0.262 *
AR (1)	0.806 *		
R-squared	0.959	0.945	0.819
Test Hausman		0.000	0.000
Test Breusch-Pagan	0.000	0.000	0.000
Durbin Watson stat	2.040	1.197	0.752
Fixed effects		Yes	Yes
dynamic effects		No	No
Observations	231	231	231

Standard errors in parentheses.

Note: Own elaboration with data from World Bank, Harvard University, Economic Commission for Latin America, 2000-2020, using Eviews 12.

** p<0.05.

* p<0.1.

To determine the best model (fixed or random effects), the Hausman Test is used, which compares the beta coefficients obtained through the fixed and random effects estimators, identifying whether the differences between them are significant. In both cases, the weight matrix is homoscedastic. Thus, considering that the Prob > Chi ^2^ is less than 5%, the fixed effects model is employed, which shows a higher global significance of 94.5%.

The interpretation of the fixed effects details that for every 1% increase in the exports of high-technology products, trade openness increased by 0.05%. Similarly, a 1% increase in exports raises trade openness by 0.30%; a 1% increase in imports leads to a 0.09% increase in trade openness; a 1% increase in total interregional trade of goods increases trade openness by 0.005%; and a 1% increase in spending on research and development results in a 0.14% increase in trade openness. Regarding gross domestic product, a 1% increase led to a 0.42% decrease in trade openness. For the economic complexity index, a 1% increase resulted in a 0.08% decrease in trade openness, and for the logistics performance index, a 1% increase led to a 0.13% decrease in trade openness during the analysis period 2000-2020.

These findings provide valuable insights into the factors that influence trade openness in South American countries, highlighting the complex interplay between various economic and logistical variables. The results underscore the importance of considering both economic structures and policy environments when assessing the openness of trade regimes.

## 6. Discussion

The impact of economic complexity, innovation, research and development, and economic growth on the trade openness of South American countries in the period 2000-2020 shows a differential behavior. These countries have exhibited lower trade openness in their economies over the last decade compared to the international market. Additionally, there is a reduced economic complexity due to lower diversification of productive matrices in export baskets, resulting in economies still heavily reliant on commodities or primary export products.

**Figure 3.**
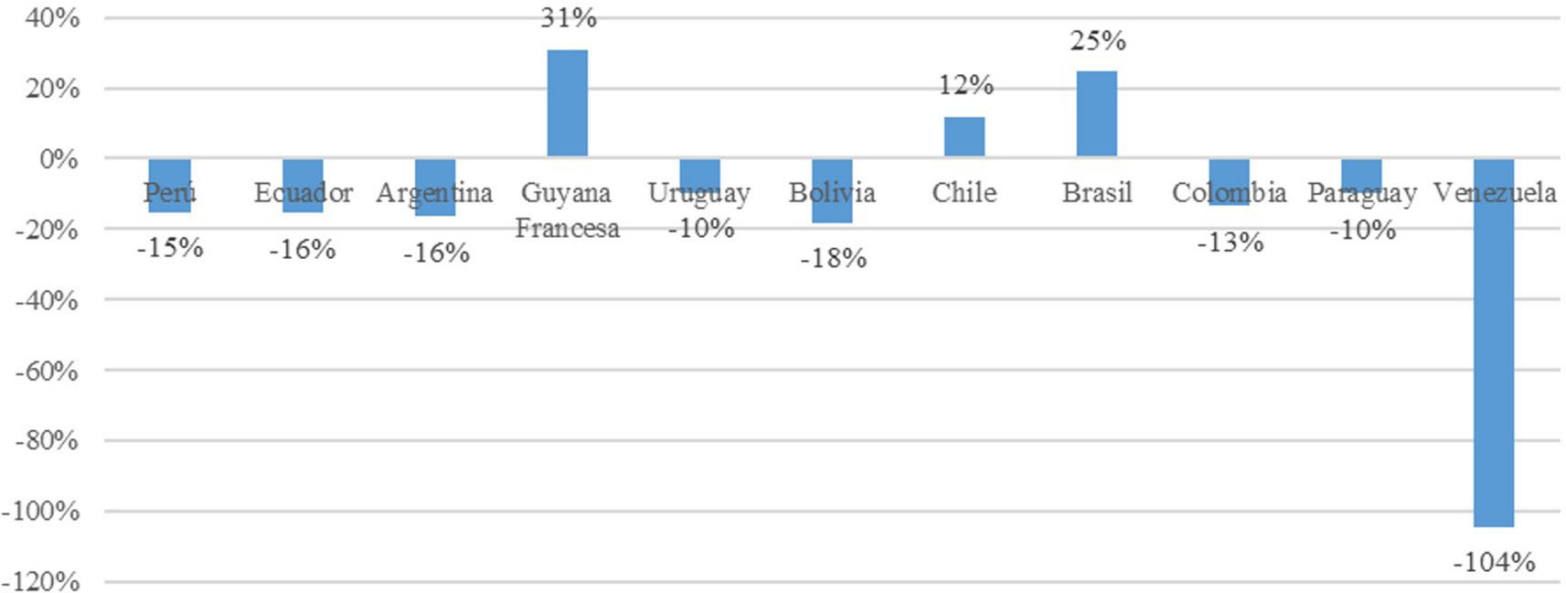
Impact on trade openness by country. Note: Own elaboration with data from World Bank (2024), Harvard University (2024), Economic Commission for Latin America (2024), for the period 2000-2020, using Eviews 12.

The greatest decline in trade openness is observed in Venezuela, with a negative impact of 104%. This is attributed to the country’s current economic crisis characterized by inflation and high prices of its oil commodity, coupled with a closed economy model. Among the remaining market economy countries, negative impacts are noted in Peru (15%), Ecuador (16%), Argentina (16%), Uruguay (10%), Bolivia had the greatest decline in Trade Openness at 18%, Ecuador and Argentina had 16%, Colombia had 13%, and Paraguay had 10%; these declines were due to diminishing returns and the accumulation of physical capital. Conversely, Chile, French Guiana, and Brazil have experienced positive impacts of 12%, 31%, and 25%, respectively. These economies are characterized by better performance in the economic complexity index, higher percentage of budget allocated to research and development expenses, and trade policies focused on industrializing value-added products such as high-tech products ( Figure 3).

These results align with the findings of the Economic Commission for Latin America and the Caribbean ( ECLAC, 2021), which has shown that economies in South America have exhibited lower trade openness due to the slowdown in international trade. Countries such as Peru, Ecuador, Bolivia, Chile, Colombia, Paraguay, and Venezuela have relied on their primary sector as the backbone of their export industry.

Furthermore, these results are consistent with the findings of Mesquita Moreira and Stein (2019), who found that while significant trade liberalization in Latin America has led to a positive impact on economic growth, challenges remain in terms of technological aspects and innovation to sustain solid international trade growth. There are still comparative advantages that allow for productivity leaps, yet South American countries have not yet taken off in terms of innovation and thus achieving higher productivity.

Corrales Sillo (2021) and Mesquita Moreira and Stein (2019) also suggest that the aforementioned results indicate that South American economies still lack knowledge accumulation, which translates into a lack of innovation. To address this, policies focusing on innovation and development are needed to add more value to export products and access larger markets, thereby enhancing competitiveness. These economies are characterized by diminishing returns due to the accumulation of physical capital.

## 7. Conclusions

In the analysis period of 2000-2020, South American countries exhibited differential behavior as they showed lower trade openness in their economies compared to the international market. Additionally, there was lower economic complexity due to less diversification in export baskets, resulting in economies still based on commodities or primary export products.

Based on panel data analysis using fixed effects, a total of 8 countries—Peru, Ecuador, Argentina, Uruguay, Bolivia, Colombia, Paraguay, and Venezuela—experienced a negative impact on trade openness. Only the economies of Chile, French Guiana, and Brazil showed a positive impact on trade openness, characterized by better performance in economic complexity indices. These countries allocate a higher percentage of their budget to research and development and pursue trade policies aimed at industrializing products with added value.

Interpretation of fixed effects reveals that the variables with the greatest impact on trade openness during 2000-2020 were gross domestic product, logistics performance index, economic complexity index, exports, imports, and research and development expenditure.

While significant trade liberalization in Latin America has led to a positive impact on economic growth, challenges remain in terms of technological aspects and innovation to sustain solid international trade growth. South American countries still struggle to innovate and achieve higher productivity, facing a slowdown in international trade despite high dependence on imports and extensive trade relations with major global economic powers amidst economic uncertainty and a shift towards left-wing political systems.

For South American countries, it is recommended to strengthen policies that promote investment in research and development to drive innovation, as well as enhance education in science, technology, engineering, and mathematics to foster creative industries that add value to primary export products.

Developing strategic investment planning for infrastructure such as roads, ports, airports, and information technologies is essential to improve connectivity and facilitate the transportation of goods and services, thereby boosting energy efficiency and reducing logistical production costs.

Identifying the positive effect of trade liberalization on economic growth in Latin American countries, albeit unsustainable, calls for the development of clusters as a key strategy to foster competitiveness and specialization in specific sectors of the economy. This could be accompanied by fiscal and financial incentive programs to encourage investment in research and development, as well as the establishment of industrial parks, shared laboratories, and innovation centers to optimize connectivity and logistics.

## Data Availability

Zenodo. The macroeconomic determinants of trade openness in Latin American countries: A panel data analysis. https://doi.org/10.5281/zenodo.13207898 ( Morán et al., 2024). This project contains the following underlying data:
• Final results of the database.xlsx This project contains the following extended data:
•
Rectoral Resolution N° 760-2007_UCV_Code Of Ethics.pdf
•

STROBE_checklist_v4_combined.pdf
•
Figure 1_Correlation of model variables •
Figure 2_Trade openness index by country •
Figure 3_Impact on trade openness by country Creative Commons Zero v1.0 Universal (CC0 License)
