# The macroeconomic determinants of trade openness in Latin American countries: A panel data analysis

**DOI:** 10.12688/f1000research.153690.4

**Published:** 2025-02-17

**Authors:** Rogger Orlando Morán Santamaría, Yefferson Llonto Caicedo, Francisco Eduardo Cúneo Fernández, Lizana Guevara Nikolays Pedro, Castro Mejía Percy Junior, Milagros Judith Pérez Pérez, Lindon Vela Meléndez, Moises Elias Montenegro López

**Affiliations:** 1 La Libertad, Universidad Cesar Vallejo, Trujillo, La Libertad, Peru; 2 Lambayeque, Universidad Nacional Pedro Ruiz Gallo, Lambayeque, Lambayeque, Peru

**Keywords:** Trade openness; impact; panel data

## Abstract

**Background:**

Trade openness shows a positive impact on economic growth, supported by economic theory, and export diversification and economic complexity show a positive dynamic in trade openness in the world; however, a specificity is generated in South American countries. Therefore, the objective of the research is to analyse the macroeconomic determinants of trade openness in Latin American countries.

**Methods:**

The research approach was quantitative and explanatory using panel data methodology from the databases of the World Bank, Harvard University and the Economic Commission for Latin America and the Caribbean for the period 2000-2020.

**Results:**

The fixed effects panel data model showed that the variables that had a negative impact on trade openness were GDP, the economic complexity index and the logistic performance index, while the variables that had a positive impact were exports of high-tech products (a proxy for innovation), exports, imports, research and development expenditure and interregional trade in goods.

**Conclusions:**

Therefore, during the analysis period of 2000-2020 in South America, based on the panel data analysis under fixed effects, a total of 8 countries had a negative impact on trade openness, and only the economies of Chile, French Guiana, and Brazil had a positive impact on trade openness; these economies are characterized by their better performance in the economic complexity index, their higher percentage of budget for research and development expenses, and their trade policies oriented towards the industrialization of their value-added products.

## 1. Introduction

The expansion of trade in countries around the world has proven to be a crucial mechanism for addressing some of the world’s most pressing crises, offering solutions to challenges like food security, which has been exacerbated by inflationary pressures. Moreover, the deepening of this trade liberalization fosters the development of key elements shaping economic complexity, such as the diversification of products that reveal comparative advantages, while also creating an environment that encourages innovation, thereby driving economic growth ( Bardi & Hfaiedh, 2021; Fusco et al., 2020; Gallego & Zofío, 2018; Tabash et al., 2022). Additionally, sustainable trade practices are essential for promoting long-term economic resilience, environmental protection, and social well-being, aligning with broader sustainable development goals.

The growing international trade flow, the development of technological capabilities, research and development, and economic complexity have had a direct and significant impact on sustainable economic growth. From the theoretical perspective of economic policy for sustainable growth, trade policy plays a fundamental role in the state, as market economies require the existence of an efficient and effective state that can ensure compliance with free trade rules ( Cetin et al., 2018; Chávez, 2021; Lima & Alvarez, 2018).

It is important to acknowledge the existence of an ongoing debate surrounding the so-called resource curse. In this regard, evidence shows that, after the 1980s and 1990s, Latin American economies developed strategies to diversify exports and foster the reorientation of external sectors, producing goods with medium to high technological complexity. Meanwhile, other countries such as Peru, Ecuador, Bolivia, Chile, Colombia, Paraguay, and Venezuela based their export industries on primary sector goods as the backbone of their commercial basket ( Alvarado & Guzmán, 2019; Gnangnon, 2022; Tabash et al., 2022).

In the South American region, according to data from the Economic Commission for Latin America (2021), the average growth in trade openness—used to assess the magnitude of foreign trade relative to a country’s domestic output—in Argentina reached 28% over the period 1990–2022. This measure has been volatile, with the highest levels of openness observed between 2002 and 2008, followed by a decline, ultimately reaching 31.7% in 2022.

In Bolivia, the average trade openness indicator was 60.1% over the 1990–2022 period, showing an upward trend from 1990 to 2008 and reaching 67.8% in 2022. In Brazil, the average trade openness growth reached 24.3% between 1990 and 2022, following an upward trend to reach 38.8% by 2022.

In Chile, trade openness averaged 62.4% from 1990 to 2022, reaching 75% in 2022; while in Colombia, the average for the same period was 35.3%, with an increase to 48.4% in 2022. Ecuador’s trade openness averaged 50.9%, reaching 57.7% in 2022. For Paraguay, the trade openness level was 82.8% during 1990–2022, while Peru reached 42.4% over the same period, and Uruguay reached 52.7%.

Arguments in favor of trade liberalization emphasize greater accumulation of physical capital, although this faces diminishing returns. Instead, the growth driver focuses on knowledge accumulation, which creates significant, dynamic impacts, leading companies that need access to large markets to innovate and become more competitive. This is due to the fluidity in their capacity to learn and assimilate new technologies ( Corrales Sillo, 2021; Mesquita & Stein, 2019).

While trade liberalization has empirically demonstrated its positive externalities in economies, clear empirical factors fully supporting this phenomenon are still lacking, particularly in Latin American economies. In this region, the impact of trade liberalization is estimated to reflect an acceleration in annual per capita GDP growth of between 0.6 and 0.7 percentage points, on average. This effect is largely due to the roles of political, institutional, and structural factors, particularly concerning technology and innovation. However, the unique characteristics of South American countries may have limited the benefits of trade liberalization, given the low levels of human capital and their distance from the technological frontier. These conditions can diminish the positive impact of trade liberalization, as economic theory suggests ( Grossman & Helpman, 2015; Goldberg & Pavcnik, 2016).

Trade liberalization has undoubtedly allowed Latin America to move beyond decades of economic stagnation. The shift in trade policy has been a key factor in the region’s development and well-being ( Herreros and Rosales, 2013; Lopez & Munoz, 2020). As Boloña (1993) held, “precisely because we are a small country in economic terms, we must diversify our markets, expand the scale of our economy, benefit from international markets to grow and accumulate capital, to overcome poverty and achieve the desired levels of well-being and prosperity” (p.13).

In this regard, Falcón (2021) points out that one of the main challenges for trade policy is to guide economies toward greater diversification of productive structures. This shift would allow countries in the region to advance toward higher economic complexity based on knowledge accumulation, moving beyond physical or labor capital alone. Such a transformation includes technological development and an evolution of the export basket toward higher value-added manufacturing, reducing dependency on commodities and generating sustainable comparative advantages. There is, therefore, a clear relationship between trade liberalization, economic complexity, innovation, research and development, and economic growth ( Lee & Lee, 2020; Shahzad et al., 2022).

Understanding the productive transformation unique to each country enables the connection of these key variables for public policy decision-making. In this way, an external sector transformation can be promoted that fosters sustainable growth with a developmental perspective, particularly in emerging economies ( Yang et al., 2023; Cheong, 2023).

In this context, the present article aims to analyze the macroeconomic determinants of trade liberalization in Latin American countries. To this end, it poses the question: What are the macroeconomic determinants of trade liberalization in Latin American countries? A panel data analysis will be employed, accounting for variables such as economic complexity, innovation, research and development, and economic growth during the period 2000–2020.

The primary contribution of this study lies in the specificity of its model, which is crucial for policymakers. By focusing exclusively on South American countries within a defined period (2000–2020), the study adds to the empirical evidence on the importance of better integration into the global economy through enhanced competitiveness. In this framework, trade liberalization is viewed as a relevant factor for income distribution and poverty reduction, promoting investment and driving international trade dynamics within the region.

The growth of international trade flows, driven by technological development and investment in R&D, has been a key factor in sustainable economic growth, especially within economies with trade liberalization policies. According to Castañeda Martínez and López González (2022) trade liberalization should promote technological diffusion and economic growth, but in some cases, such as Mexico, the expected effects do not materialize due to structural limitations. However, Grande González (2023) highlights that innovation and digitalization have significantly improved the competitiveness of developed economies, provided that appropriate regulatory frameworks are in place. In this context, economic policies aimed at trade liberalization must be accompanied by investment strategies in technological infrastructure and the strengthening of the R&D ecosystem to ensure that the benefits of trade are not concentrated in just a few sectors and economic actors ( Orquera Carranco, 2023).

Moreover, the impact of trade agreements on attracting foreign investment reinforces the relationship between trade openness and economic development. Castañeda Martínez and López González (2022) how treaties such as NAFTA have increased foreign direct investment in Mexico, strengthening its industry and economic growth. However, Orquera Carranco (2023) emphasizes that companies must play an active role in sustainable development, aligning their strategies with the Sustainable Development Goals (SDGs) to ensure equitable economic growth. In this regard, Castañeda Martínez and López González (2022) highlight the role of international organizations, such as the OECD, in promoting free trade and fiscal regulation, though challenges remain regarding the fairness of global governance. Thus, trade liberalization will only be effective in terms of sustainability if complemented by policies that strengthen technology, incentivize research, and promote inclusive development strategies.

However, a knowledge gap remains regarding the specific macroeconomic determinants influencing trade liberalization in Latin America. Despite evidence suggesting that factors like economic complexity and innovation play significant roles, it is not entirely clear how these factors interact with other structural and political aspects unique to the region. This study seeks to address that gap by providing a detailed analysis that can serve as a foundation for more effective public policy formulation across the region.

## 2. Literature review

Recent empirical evidence underscores the relevance of the external sector, driven by trade liberalization policies, as a key factor in the growth of emerging economies in Latin America, especially amid current health, food security, and economic uncertainty crises. This context has highlighted the need to diversify and enhance the complexity of exports, prompting the restructuring of the productive matrix. This transformation demands not only the accumulation of physical capital and expansion of capacities but also the development of an innovation-friendly business environment and increased investment in research and development ( Carrasco, 2021; Chávez, 2021; Bashir et al., 2020; Chacha et al., 2024; Guo et al., 2023; Mania & Rieber, 2019; Nieminen, 2020).

In the international context, Wang et al. (2023) conduct a study evaluating economic complexity, trade diversification, renewable energy consumption, and environmental taxes as drivers of growth from 1995 to 2018. The findings indicate that these factors significantly promoted growth in BRICTS countries, with relevant policy implications for achieving marginal contributions to economic development. Similarly, Li and Rigby (2023) find a positive and significant relationship between economic growth and complex technological diversification in China, specifically in 1991-1995 and 2011-2015. Here, knowledge complexity and smart specialization demonstrate a knowledge development pathway toward achieving regional competitive advantage.

A panel VAR analysis across 11 European countries (2001-2016) reveals a unidirectional relationship between economic growth and financial development, and between trade and economic growth, but a negative association between innovation and economic growth, as well as between trade and innovation ( Mtar & Belazreg, 2023). This suggests that each country’s role depends on innovation and productivity, and recommends that institutional quality is essential for improving trade regulation and fostering innovation in local companies to mitigate the effects of trade liberalization.

Various studies demonstrate relationships between economic complexity, innovation, research and development, and economic growth. In 28 African countries (1995-2019), analysis shows how economic complexity moderates the impact of natural resources on economic growth in both the short and long term, proposing policies to effectively link these factors ( Mesagan & Vo, 2023). In Russia, the creation of high-tech products and markets is viewed as essential for long-term economic development, where technological diversification in high-tech sectors drives competitiveness and market expansion ( Goryushkin & Khalimova, 2023).

Additionally, research shows that export diversification and economic complexity positively correlate with per capita growth, which influences GDP and economic vulnerability, especially in countries facing challenges such as low growth, high unemployment, rising debt, and regional vulnerability ( Ben Saad et al., 2023). Rahman et al. (2023) in Bangladesh highlight that technological innovation, foreign investment, trade, and human capital have both short- and long-term associations with economic growth. Bardi and Hfaiedh (2021) also demonstrate that trade liberalization positively impacts economic growth, supported by classical theories ( Barro & Sala, 2009; Lucas, 1988; Romer, 1986; Banday et al., 2021; Zaman et al., 2021).

Canh and Thanh (2022) for their part, evidence that the diversification of exports and economic complexity show a positive dynamic, presenting bidirectional causality given the role played by the quality of export products which is measured from the productive capacities of a country to add value to its export products achieving more competitiveness through the structural transformation of economies and the diversification of the productive matrix of countries ( Armas et al., 2020; Shahzad et al., 2022; Lapatinas et al., 2019; Koch, 2021).

While Mesquita and Stein (2019) they consider that although the great trade liberalization in Latin America has led to a positive impact on economic growth and well-being being the channel that helped recover much of the economies of Latin America from their participation in trade in the gross domestic product in the region that reached 28% they still face challenges in terms of technological aspect and innovation to continue the solid growth of international trade; this because there are comparative advantages that allow productivity leaps ( Llanos & Brown, 2021; Vela et al., 2021).

For Chávez (2021) considers in the panel data model two related equations innovation as the dependent variable and trade openness as the independent variable and as a second equation was obtained as the dependent variable to the predictions of innovation and trade openness as the explanatory variable.

Meanwhile, Fusco et al. (2020) considers in their analysis of dynamic panel data where they analyze food security and trade openness developing three regression equations with indicators of trade openness, tariffs, and globalization. While Cetin et al. (2018) considers economic growth, energy consumption, trade openness, and financial development as explanatory variables and carbon emissions as the dependent variable for the case of Turkey.

## 3. Theoretical basis

### 3.1 Trade openness

Gräbner et al. (2021) believe that international trade generates economic growth, suggesting that while trade openness is not a direct or robust determinant in economic growth, it contributes significantly to it. By leveraging growth potential, greater trade openness generates ideas and knowledge, along with their respective technical advancements, to open up new goods and services in international trade, contributing from the theory of the openness paradigm and initiating a debate on the causality of growth ( Silajdzic et al., 2018).

### 3.2 Economic growth

Hartmann (2014) from the contribution of economic theory, considers that growth is linked to the increase in production, income, and consumption. Meanwhile, the classic view of Smith (1976) considers that both the division of labor and trade are key aspects to achieving growth, explained by the classical factors of capital, labor, and land, but with an additional factor related to technology ( Solow, 1956). Subsequently, approaches to economic growth explained by technological progress emerged, providing a different perspective on long-term growth ( Romer, 1986) with technology as an exogenous factor of growth rather than an endogenous one.

### 3.3 Economic complexity

Hausmann et al. (2020) consider the theory of economic complexity, which involves moving away from traditional factors to focus on knowledge as the main value generator for achieving a new structural transformation that allows diversifying the productive matrix and empowering the aspect of greater competitiveness. For Hidalgo and Hausmann (2009) economic complexity refers to the set of a region’s export products that shows a country’s productive capacity based on existing capabilities and know-how, implying that the more diverse and value-added the export products, the more productive the region is considered.

### 3.4 Innovation

The paradigm of innovation and structural changes have become prominent in recent literature due to the composition of new economic systems where creation, innovation, and entrepreneurship have led to major changes in countries like India, China, and the United States, whose knowledge-based growth has led to their exponential development. Schumpeter (1941) in his theory of economic development addressed that innovation, as well as inventions by scientists, would lead to creating entirely new investment, growth, and employment opportunities. According to Schumpeter and Swedberg (2003) the innovation process can be divided into four dimensions: invention, innovation, diffusion, and imitation.

### 3.5 Research and development

Elfaki and Ahmed (2024) consider that as a result of technological adoption, these elements are fundamental for the economic growth of any nation. In a world where global competition is increasingly intense, the ability to innovate and continuously improve becomes a determining factor for economic success. R&D drives progress, not only through the creation of new products and services but also by optimizing existing processes, thus increasing productivity and efficiency across various industries.

Companies that allocate resources to research and development tend to be at the forefront of creating novel products and services that better meet consumer needs. This not only makes them more competitive but also enables the creation of new industries and markets, which in turn can lead to sustained economic growth.

Being the hypothesis that economic complexity, innovation, research and development, and economic growth are the determinants of trade openness.

## 4. Methods

### 4.1 Methodological design

A quantitative approach is considered, with a type of explanatory research that corresponds to identifying the impact of economic complexity, innovation, research and development, and economic growth on the trade openness of South American countries during the period 2000-2020.

The variables analyzed have not been modified, and the research is a non-experimental design; therefore, it has been verified that it was conducted in a specific context of the South American countries and the interaction of the unmanipulated variables ( Hernández et al., 2018).

The use of a panel data model in this research is statistically robust and methodologically relevant for several reasons. First, this approach allows for controlling unobservable heterogeneity among the South American countries analyzed, which is crucial since each country has unique characteristics that may influence trade openness, such as institutional, cultural, or geographical differences. By incorporating fixed or random effects, the panel model minimizes bias due to omitted variables, improving the accuracy of estimates.

Moreover, by combining cross-sectional data (across countries) and temporal data (over 21 years), the model leverages greater variability in the data, enhancing statistical efficiency and the ability to identify causal relationships. The choice of this method is also supported by economic literature, which has demonstrated its usefulness in analyzing similar dynamics in international contexts ( Barro, 1990; Romer, 1990).

Finally, the robustness of the model is reinforced by the use of data from reliable and well-recognized sources, such as the World Bank and Economic Commission for Latin America and the Caribbean (ECLAC), ensuring the quality and consistency of the information. Altogether, these characteristics ensure that the panel data model is a solid and appropriate tool for examining the macroeconomic determinants of trade openness in South American countries ( World Bank, 2024; Economic Commission for Latin America, 2024).

### 4.2 Population and sampling

The population involves a total of 2079 data points across 9 analyzed variables considering a total of 11 South American countries, noting that we have 231 data points per variable for each country under analysis during the 2000-2020 period.

The sample is equal to the population and comprises a total of 2079 data points across 9 analyzed variables considering a total of 11 South American countries, noting that we have 231 data points per variable for each country under analysis during the 2000-2020 period.

The unit of analysis consists of each South American country, involving a total of 11 countries analyzed during the 2000-2020 period.

### 4.3 Data collection technique and procedure

For measuring the variables, documentary analysis was used as the technique, and the observation guide served as the instrument, which is a tool used to collect data systematically and structured during an observation or study.

The data obtained from the World Bank corresponded to the explanatory variables of gross domestic product, imports, exports, high-tech product exports, research and development expenditure, and the logistics performance index; as well as from the Harvard University Economic Complexity Atlas, where information on the economic complexity index was obtained. Regarding the interregional trade of goods variable, it was obtained from the database of the Economic Commission for Latin America and the Caribbean during the mentioned analysis period.

A panel data model is used, where from this model, the impact of economic complexity, innovation, research and development, and economic growth on the trade openness of the South American countries during the 2000-2020 period is quantified. To perform the estimates consistently and achieve a proper analysis, the variables have been transformed into logarithms such as gross domestic product, imports, exports, high-tech product exports, and total interregional trade of goods.

### 4.4 Data analysis

The databases used for this research stem from international institutions such as the World Bank, Harvard University, and the Economic Commission for Latin America for the period 2000-2020.

In the estimation, we have used the panel data model based on economic literature that demonstrates a positive impact of trade openness on economic growth ( Romer, 1986; Barro & Sala, 2009; Lucas, 1988; Katz, 1999).

Highlighting the importance of export diversification and complexity, leading to restructuring the production matrix, which in turn requires the accumulation of phys-ical capital and expansion of capacities, thereby necessitating the development of a climate of entrepreneurial innovation, as well as allocating greater resources to research and development ( Carrasco, 2021; Chávez, 2021).

Equation 1 establishes the relationship of the dependent variable trade openness with exports, imports, and gross domestic product per country in South America i =1, 2, …………. 11 in the year t = 2000-2020.

ACi,t=Bo+B1(Exporti,t)+B2(Importi,t)+B3(Pbii,t)+ei,t (1)

On the other hand, the incorporation of the variable’s economic complexity (eco-nomic complexity index), innovation (proxy: exports of high-tech products), research and development (percentage expenditure on research and development), the logistic performance index, and total interregional trade of goods are evidenced in the following equation:

ACi,t=Bo+B1(Exporti,t)+B2(Importi,t)+B3(Pbii,t)+B4(ICEi,t)+B5(Export_teci,t)+B6(i_di,t)+B7(i_logi,t)+B8(c_interi,t)+ei,t (2)

Next, the Generalized Least Squares (GLS) model is estimated because the equations present problems of heteroskedasticity and autocorrelation detected with the Breusch and Pagan (1979) (as cited in Onali et al., 2017) and Wooldridge (2010) Lagrange Multiplier tests. Furthermore, to choose between a fixed or random effects model, the Hausman test (1978) (as cited in Onali et al., 2017) is utilized.

The variables used in the proposed panel data model are detailed in the following Table 1.

**Table 1.** Variables used in the model.

Variable	Description	Source
Trade openness (ac i,t)	Economic openness index measuring the degree of a country's economic openness; calculated as the sum of exports plus imports divided by gross domestic product.	World Bank 2000-2020
Exports (Export i,t)	Merchandise exports (US$ at current prices)	World Bank 2000-2020
Imports (Import i,t)	Merchandise imports (US$ at current prices)	World Bank 2000-2020
Gross Domestic Product (Pbi i,t)	Value of all goods and other market services exported to the rest of the world by department, US$ at current prices	World Bank 2000-2020
Export of High-Tech Products (Export_tec i,t)	Value of exports of high-tech products, which are highly research and development intensive products	World Bank 2000-2020
Research and Development Expenditure (i_d i,t)	Expenditure on research and development (% of GDP)	World Bank 2000-2020
Logistic Performance Index (i_log i,t)	Logistic Performance Index: Total (From 1=low to 5=high)	World Bank 2000-2020
Economic Complexity Index (ice i,t)	Economic Complexity Index, a set of export products from a region showing a country's productive capacity based on existing capabilities and know-how	Harvard University, 2000-2020
Total Interregional Trade of Goods (c_inter _i,t_)	Value of trade in goods between countries, FOB exports in millions of dollars	Economic Commission for Latin America, 2000-2020

Note: Own elaboration with data from World Bank, Harvard University, Economic Commission for Latin America, 2000-2020, using Eviews 12.

**Figure 1.**
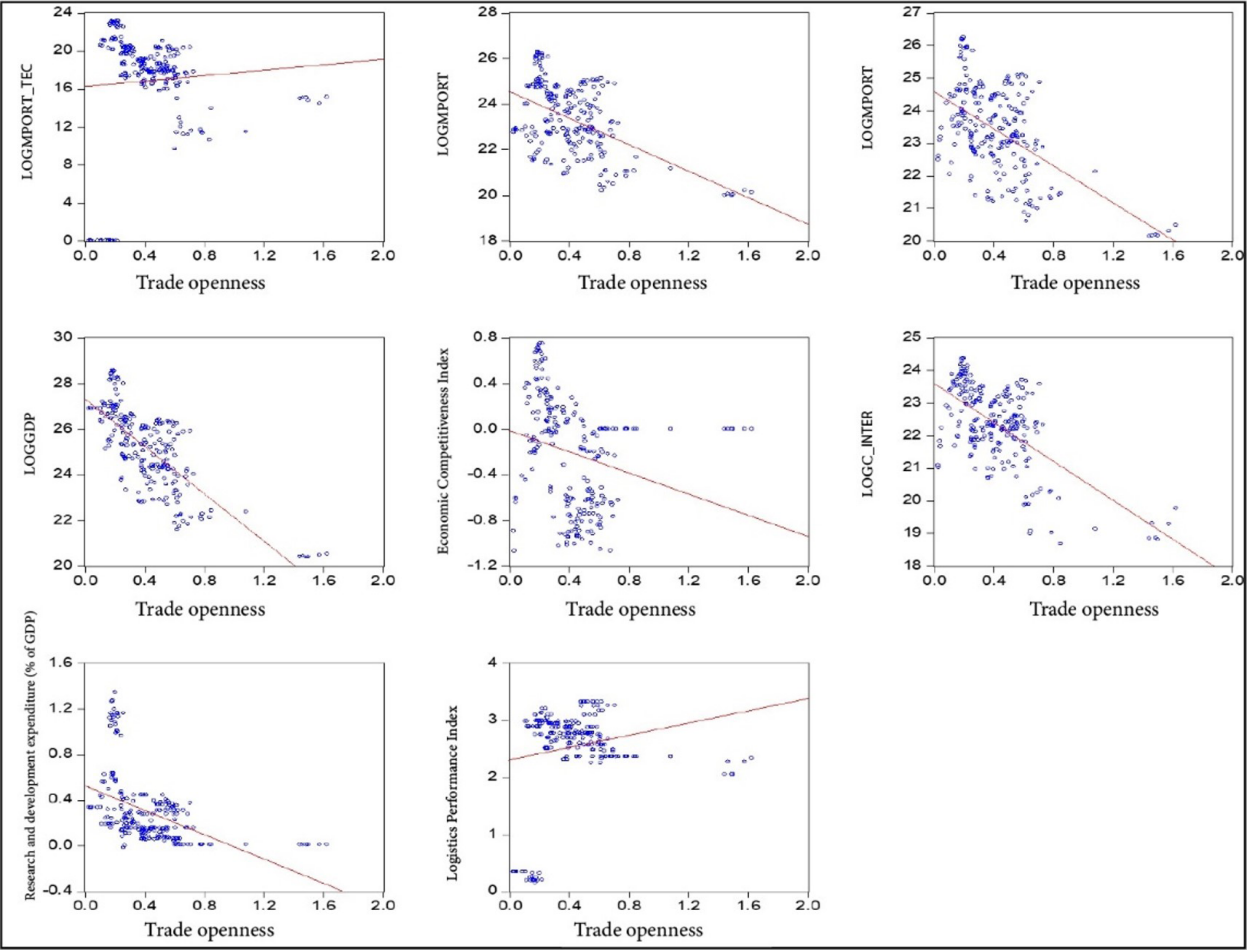
Correlation of model variables. Note: Own elaboration with data from World Bank (2024), Harvard University (2024), Economic Commission for Latin America (2024), for the period 2000-2020, using Eviews 12.

The data were analyzed using an econometric panel data model that captures the bidimensionality of the data. This model allows for the quantification of the impact of economic complexity, innovation, research and development, and economic growth on the trade openness of South American countries during the period 2000-2020. The data were obtained from the World Bank, Harvard University, and the Economic Commission for Latin America and the Caribbean and processed in Eviews 12 and is used for the estimation of the panel data model, considering the control variables that are subjected to the proposed equations to analyze the interrelationship between the variables ( Figure 1). This allows for both descriptive statistical treatment and the estimation of the proposed panel data model and the Generalized Least Squares (GLS) model. An academic license for the use of Eviews 12 software is available, registered under the name Lindon Vela Meléndez. The license details are as follows: Serial number: Q1208886 - D49010AF - 9D854485. The software can be downloaded from the following link: http://www.eviews.com/download/student12.

Furthermore, the Generalized Least Squares (GLS) model was employed due to issues of heteroscedasticity and autocorrelation identified in the structural equations. These issues were detected using the Breusch and Pagan (1979) (as cited in Onali et al., 2017) Lagrange Multiplier test and the Wooldridge (2010) test. Additionally, the Hausman (1978) (as cited in Onali et al., 2017) test was used to decide between a fixed or random effects model, which helps in choosing the most suitable model based on the consistency of the estimators.

### 4.3 Ethical considerations

The data for this study were obtained from international institutions such as the World Bank, Harvard University and the Economic Commission for Latin America for the period 2000-2020. Access to the data is public and free of charge on the detailed websites and adheres to rigorous ethical standards to protect the confidentiality of the published data.

In addition, the data from the World Bank, Harvard University and the Economic Commission for Latin America is an effort by international institutions to generate databases that allow interaction with public policy makers.

Although we cannot provide specific details on the process of obtaining the data, it is imperative to indicate that, as prestigious governmental institutions such as the World Bank, Harvard University and the Economic Commission for Latin America, there is a use and policies to access under ethical practices in the generation of data in the respective public repositories and free access and obtaining informed consent.

For the ethical use of public data obtained by the World Bank, Harvard University and the Economic Commission for Latin America, we adhere to the principles of intellectual honesty, truthfulness, transparency, human integrity, respect for intellectual property, justice and responsibility, the study aims to comply with the “Code of Ethics in Research of the Universidad César Vallejo, version 01; by Resolution of the University Council N° 0340-2021-UCV”, code of ethics that was used throughout the development of the scientific article, since as main author we work all scientific activity respecting the code of ethics of the UCV university.

We have used the data from the described repositories for research purposes only, as permitted by the conditions of use by the World Bank, Harvard University and the Economic Commission for Latin America. We have not attempted to modify any data, and our analysis does not include any personally identifiable information.

By using publicly available anonymized data and adhering to ethical principles in our research, we aim to minimize any potential ethical issues while leveraging valuable information for the benefit of research in economic science.

## 5. Results

**Figure 2.**
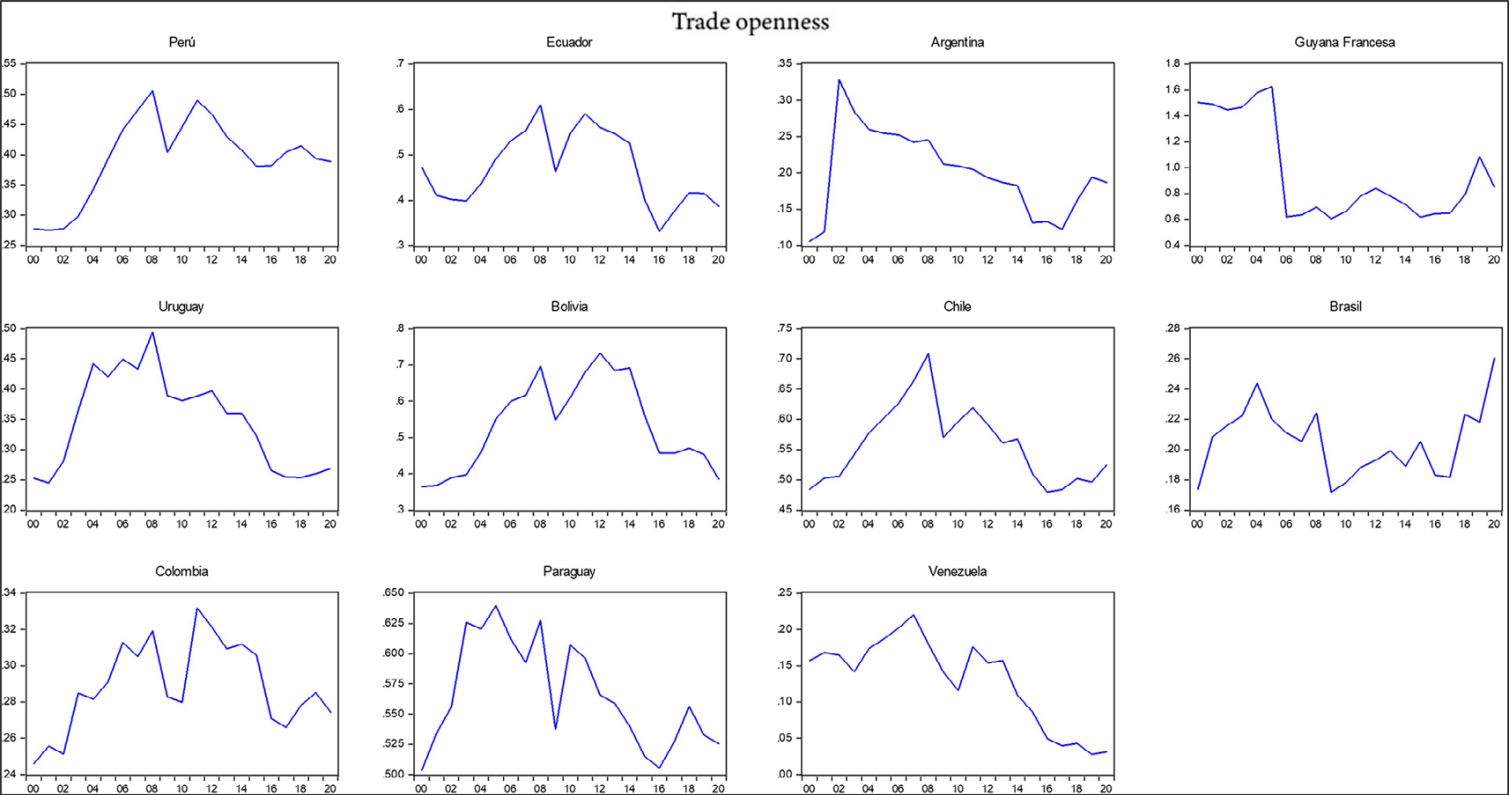
Trade openness index by country. Note: Own elaboration with data from World Bank (2024), Harvard University (2024), Economic Commission for Latin America (2024), for the period 2000-2020, using Eviews 12.

The research utilizes data sourced from the World Bank, Harvard University, and the Economic Commission for Latin America and the Caribbean. Descriptive statistics for each variable analyzed are presented in Table 2, including the mean, standard deviation, minimum, maximum, skewness, and kurtosis. Additionally, the correlation matrix between the variables under analysis is provided. It shows that exports, imports, gross domestic product (GDP), the economic complexity index, total interregional trade of goods, and research and development had a negative relationship with trade openness, whereas the export of high-technology products and the logistics performance index had a positive correlation with trade openness during the period 2000-2020. The negative relationship indicates that South American countries, except for Chile and Brazil, have shown less trade openness in their economies over the last decade with respect to the international market. According to the Economic Commission for Latin America (2021) economies in South America have demonstrated reduced trade openness due to the slowdown in international trade despite their high dependence on imports and extensive trade relations with major global economic powers in a scenario of economic uncertainty and a political shift to the left, which helps to understand which variables are influencing reduced trade openness ( Figure 2).

**Table 2.** Descriptive statistics of the model variables.

	AC	Export_tec	Export	Import	Pbi	ICE	C_INTER	I_d	I_LOG
Mean	0.42	16.87	23.32	23.38	25.13	-0.21	22.34	0.30	2.54
Median	0.39	18.31	23.23	23.35	25.32	-0.10	22.40	0.20	2.76
Maximum	1.63	23.13	26.27	26.25	28.59	0.75	24.39	1.34	3.32
Minimum	0.03	0.00	19.99	20.15	20.38	-1.07	18.68	-0.02	0.15
Std. Dev.	0.26	5.93	1.50	1.34	1.76	0.48	1.22	0.30	0.77
Skewness	1.99	-1.99	-0.15	-0.22	-0.45	0.01	-0.91	1.79	-2.24
Kurtosis	9.47	6.18	2.37	2.70	3.00	1.99	3.74	5.68	7.14
AC	1.00	0.06	-0.50	-0.54	-0.76	-0.25	-0.63	-0.46	0.18
Export_tec	0.06	1.00	0.55	0.26	0.19	0.25	0.30	0.36	0.95
Export	-0.50	0.55	1.00	0.90	0.90	0.33	0.88	0.71	0.45
Import	-0.54	0.26	0.90	1.00	0.92	0.29	0.90	0.65	0.15
Pbi	-0.76	0.19	0.90	0.92	1.00	0.37	0.91	0.72	0.06
ICE	-0.25	0.25	0.33	0.29	0.37	1.00	0.27	0.58	0.18
C_inter	-0.63	0.30	0.88	0.90	0.91	0.27	1.00	0.61	0.17
I_d	-0.46	0.36	0.71	0.65	0.72	0.58	0.61	1.00	0.22
I_LOG	0.18	0.95	0.45	0.15	0.06	0.18	0.17	0.22	1.00
N	231	231	231	231	231	231	231	231	231

Abbreviations used: “N” = Number of observations, “Std. Dev.” = Standard Deviation, “Minimum” = Minimum, “Maximum” = Maximum.

Note: Own elaboration with data from World Bank, Harvard University, Economic Commission for Latin America, 2000-2020, using Eviews 12.

The standard deviation indicates that there is greater variability among the variables of export of high-technology products, exports, imports, gross domestic product, and total interregional trade of goods, with a total of N data points equivalent to 231 observed data per variable representing the total number of 2079 observations across the 9 variables studied during the period 2000-2020 for the 11 South American countries. This variability and the detailed analysis of the correlations provide insights into the complexities of trade dynamics and economic interactions in the region, underscoring the nuanced impact of various economic factors on trade openness.

The estimated regression results, considering the Wooldridge (2010) test, indicate the presence of autocorrelation across the panels. Additionally, the Breusch and Pagan (1979) (as cited in Onali et al., 2017) Lagrange Multiplier test shows that the estimations had issues with heteroscedasticity; these issues have been corrected using Generalized Least Squares (GLS) to address both heteroscedasticity and autocorrelation.

Table 3 displays the results of the panel data estimation, revealing that public health spending, rural population, unemployment, and gross domestic product are statistically significant at the 5% level considering equation 2.

**Table 3.** Regression: Trade openness with explanatory variables.

Explanatory variable	Levels	Fixed effects	Mobile effects
Constant	1.341 *	1.439 *	2.091 *
Log Export_tec	-0.017	0.053 *	0.003
Log Export	0.243 *	0.301 *	0.379 *
Log Import	0.175 *	0.086 *	0.098 *
Log Pbi	-0.425 *	-0.425 *	-0.461 *
ICE	-0.002	-0.078 **	0.057 *
Log C_INTER	0.016	0.005	-0.027
I_d	0.130	0.141 **	0.083
I_LOG	-0.045	-0.127 *	-0.262 *
AR (1)	0.806 *		
R-squared	0.959	0.945	0.819
Test Hausman		0.000	0.000
Test Breusch-Pagan	0.000	0.000	0.000
Durbin Watson stat	2.040	1.197	0.752
Fixed effects		Yes	Yes
dynamic effects		No	No
Observations	231	231	231

Standard errors in parentheses.

Note: Own elaboration with data from World Bank, Harvard University, Economic Commission for Latin America, 2000-2020, using Eviews 12.

** p<0.05.

* p<0.1.

To determine the best model (fixed or random effects), the Hausman Test is used, which compares the beta coefficients obtained through the fixed and random effects estimators, identifying whether the differences between them are significant. In both cases, the weight matrix is homoscedastic. Thus, considering that the Prob > Chi ^2^ is less than 5%, the fixed effects model is employed, which shows a higher global significance of 94.5%.

The interpretation of the fixed effects details that for every 1% increase in the exports of high-technology products, trade openness increased by 0.05%. Similarly, a 1% increase in exports raises trade openness by 0.30%; a 1% increase in imports leads to a 0.09% increase in trade openness; a 1% increase in total interregional trade of goods increases trade openness by 0.005%; and a 1% increase in spending on research and development results in a 0.14% increase in trade openness. Regarding gross domestic product, a 1% increase led to a 0.42% decrease in trade openness. For the economic complexity index, a 1% increase resulted in a 0.08% decrease in trade openness, and for the logistics performance index, a 1% increase led to a 0.13% decrease in trade openness during the analysis period 2000-2020.

These findings provide valuable insights into the factors that influence trade openness in South American countries, highlighting the complex interplay between various economic and logistical variables. The results underscore the importance of considering both economic structures and policy environments when assessing the openness of trade regimes.

## 6. Discussion

The impact of economic complexity, innovation, research and development, and economic growth on the trade openness of South American countries in the period 2000-2020 shows a differential behavior. These countries have exhibited lower trade openness in their economies over the last decade compared to the international market. Additionally, there is a reduced economic complexity due to lower diversification of productive matrices in export baskets, resulting in economies still heavily reliant on commodities or primary export products.

**Figure 3.**
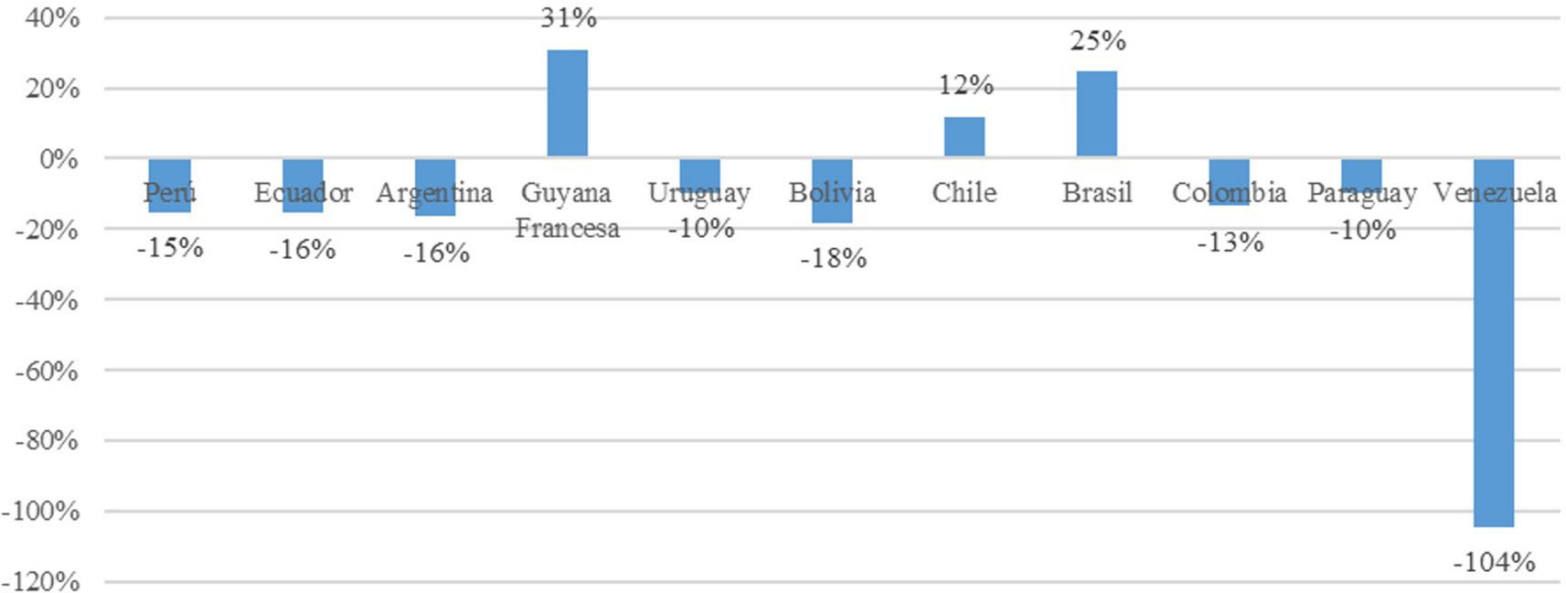
Impact on trade openness by country. Note: Own elaboration with data from World Bank (2024), Harvard University (2024), Economic Commission for Latin America (2024), for the period 2000-2020, using Eviews 12.

The greatest decline in trade openness is observed in Venezuela, with a negative impact of 104%. This is attributed to the country’s current economic crisis characterized by inflation and high prices of its oil commodity, coupled with a closed economy model. Among the remaining market economy countries, negative impacts are noted in Peru (15%), Ecuador (16%), Argentina (16%), Uruguay (10%), Bolivia had the greatest decline in Trade Openness at 18%, Ecuador and Argentina had 16%, Colombia had 13%, and Paraguay had 10%; these declines were due to diminishing returns and the accumulation of physical capital. Conversely, Chile, French Guiana, and Brazil have experienced positive impacts of 12%, 31%, and 25%, respectively. These economies are characterized by better performance in the economic complexity index, higher percentage of budget allocated to research and development expenses, and trade policies focused on industrializing value-added products such as high-tech products ( Figure 3).

These results align with the findings of the Economic Commission for Latin America and the Caribbean, which has shown that economies in South America have exhibited lower trade openness due to the slowdown in international trade. Countries such as Peru, Ecuador, Bolivia, Chile, Colombia, Paraguay, and Venezuela have relied on their primary sector as the backbone of their export industry ( Economic Commission for Latin America, 2021).

Venezuela muestra datos atípicos en el análisis de apertura comercial debido a su profunda crisis económica y su modelo de economía cerrada. A diferencia de otros países sudamericanos, Venezuela experimentó una caída del 104% en su apertura comercial, la más pronunciada en la región. Este comportamiento se explica por su dependencia extrema del petróleo como principal commodity, combinada con una inflación descontrolada y políticas económicas que han limitado la diversificación productiva y la integración con mercados internacionales. Mientras otros países, como Chile y Brasil, han logrado impactos positivos gracias a una mayor complejidad económica y políticas orientadas a la industrialización y la innovación, Venezuela ha visto un retroceso significativo en su capacidad para competir en el mercado global. Esta situación resalta la importancia de políticas comerciales y económicas que fomenten la diversificación y la inversión en investigación y desarrollo para evitar la vulnerabilidad asociada a la dependencia de recursos primarios.

Furthermore, these results are consistent with the findings of Mesquita Moreira and Stein (2019), who found that while significant trade liberalization in Latin America has led to a positive impact on economic growth, challenges remain in terms of technological aspects and innovation to sustain solid international trade growth. There are still comparative advantages that allow for productivity leaps, yet South American countries have not yet taken off in terms of innovation and thus achieving higher productivity.

Corrales Sillo (2021) and Mesquita Moreira and Stein (2019) also suggest that the aforementioned results indicate that South American economies still lack knowledge accumulation, which translates into a lack of innovation. To address this, policies focusing on innovation and development are needed to add more value to export products and access larger markets, thereby enhancing competitiveness. These economies are characterized by diminishing returns due to the accumulation of physical capital.

## 7. Conclusions

In the analysis period of 2000-2020, South American countries exhibited differential behavior as they showed lower trade openness in their economies compared to the international market. Additionally, there was lower economic complexity due to less diversification in export baskets, resulting in economies still based on commodities or primary export products.

Based on panel data analysis using fixed effects, a total of 8 countries—Peru, Ecuador, Argentina, Uruguay, Bolivia, Colombia, Paraguay, and Venezuela—experienced a negative impact on trade openness. Only the economies of Chile, French Guiana, and Brazil showed a positive impact on trade openness, characterized by better performance in economic complexity indices. These countries allocate a higher percentage of their budget to research and development and pursue trade policies aimed at industrializing products with added value.

Interpretation of fixed effects reveals that the variables with the greatest impact on trade openness during 2000-2020 were gross domestic product, logistics performance index, economic complexity index, exports, imports, and research and development expenditure.

While significant trade liberalization in Latin America has led to a positive impact on economic growth, challenges remain in terms of technological aspects and innovation to sustain solid international trade growth. South American countries still struggle to innovate and achieve higher productivity, facing a slowdown in international trade despite high dependence on imports and extensive trade relations with major global economic powers amidst economic uncertainty and a shift towards left-wing political systems.

For South American countries, it is recommended to strengthen policies that promote investment in research and development to drive innovation, as well as enhance education in science, technology, engineering, and mathematics to foster creative industries that add value to primary export products.

Developing strategic investment planning for infrastructure such as roads, ports, airports, and information technologies is essential to improve connectivity and facilitate the transportation of goods and services, thereby boosting energy efficiency and reducing logistical production costs.

Identifying the positive effect of trade liberalization on economic growth in Latin American countries, albeit unsustainable, calls for the development of clusters as a key strategy to foster competitiveness and specialization in specific sectors of the economy. This could be accompanied by fiscal and financial incentive programs to encourage investment in research and development, as well as the establishment of industrial parks, shared laboratories, and innovation centers to optimize connectivity and logistics.

The results of the panel data analysis for the period 2000–2020 reveal that trade openness in South American countries is influenced by factors such as economic complexity, innovation, research and development (R&D) spending, and economic growth. However, the findings show that most countries in the region have experienced a decline in trade openness, with the exception of Chile, Brazil, and French Guiana, which have maintained a positive impact thanks to policies focused on industrialization and export diversification. In this context, it is crucial to propose specific policy recommendations tailored to each country's realities to strengthen their integration into the global market and promote sustainable economic growth.

**a. Promotion of Research and Development (R&D):**

• **Chile and Brazil:** Both countries have demonstrated outstanding performance in R&D, contributing to greater trade openness. It is recommended to continue strengthening tax incentives for companies investing in innovation, as well as increasing collaboration between universities, research centers, and the private sector. Competitive grant programs for R&D projects and the creation of technology parks could further expand their impact.
• **Argentina and Uruguay:** These countries have a solid scientific foundation but require greater investment in R&D. Policies should encourage technology transfer from academic sectors to industry and the creation of specialized technology clusters in sectors such as biotechnology and agribusiness.
• **Peru, Ecuador, and Bolivia:** With economies still dependent on commodities, it is essential to diversify R&D investment into higher value-added sectors. Training programs in advanced technologies and the creation of public funds to finance innovation projects in small and medium-sized enterprises (SMEs) could be effective strategies.

**b. Improvement of Logistics Infrastructure:**

• **Colombia and Paraguay:** Both countries face transportation infrastructure challenges that limit their ability to integrate into global value chains. Investment should be prioritized in logistics corridors connecting ports, airports, and production centers, as well as modernizing customs procedures to streamline international trade.
• **Venezuela:** Given its economic crisis, the urgent rehabilitation of basic infrastructure, such as roads and ports, is necessary with the support of international organizations. Technical and financial cooperation programs could facilitate the reconstruction of its logistical capacity.

**c. Export Diversification:**

• **Chile and Brazil:** These countries have successfully diversified their exports into high-tech products. It is recommended to continue promoting sectors such as sustainable mining, renewable energy, and advanced manufacturing through tax incentives and access to international markets.
• **Argentina and Uruguay:** With a strong agro-industrial base, these countries could diversify into organic and niche products, capitalizing on growing global demand. Certification policies and international promotion efforts would be key.
• **Peru, Ecuador, and Bolivia:** Reducing dependence on commodities is crucial by promoting sectors such as tourism, non-traditional agro-exports, and light manufacturing. Training programs in business management and access to financing for exporting SMEs could be decisive.

**d. Strengthening Education in Science and Technology:**

• **All countries:** The development of specialized human capital is essential to drive innovation and competitiveness. It is recommended to increase investment in STEM education (science, technology, engineering, and mathematics) and promote partnerships between educational institutions and the productive sector to ensure that academic programs align with labor market needs.

**e. Creation of Clusters and Industrial Parks:**

• **Chile, Brazil, and Argentina:** These countries could lead the creation of industrial clusters specialized in sectors such as green mining, biotechnology, and Industry 4.0. Public-private collaboration and attracting foreign direct investment (FDI) would be key to their success.
• **Peru, Ecuador, and Bolivia:** The formation of clusters in sectors such as agribusiness and tourism could generate economies of scale and improve international competitiveness.

In conclusion, the implementation of these specific recommendations, tailored to each country's realities, would not only enhance trade openness but also promote sustainable and diversified economic growth in the region. Collaboration between governments, the private sector, and international organizations will be key to achieving these goals.

## Data Availability

This section presents the original database and the extended database, including calculations and figures derived from them, for replication purposes by researchers and readers in general. Zenodo. The macroeconomic determinants of trade openness in Latin American countries: A panel data analysis. https://doi.org/10.5281/zenodo.14835488 ( Morán et al., 2024). This project contains the following underlying data:
• Final results of the database.xlsx • Data of the variables used in the model • Results of the regression of commercial openness with explanatory variables This project contains the following extended data:
• Rectoral Resolution N° 760-2007_UCV_Code Of Ethics.pdf • STROBE_checklist_v4_combined.pdf •
Figure 1_Correlation of model variables •
Figure 2_Trade openness index by country •
Figure 3_Impact on trade openness by country Creative Commons Zero v1.0 Universal (CC0 License)
