# Supplementary material for: The macroeconomic determinants of trade openness in Latin American countries: A panel data analysis
Source: F1000Res. 2025 Feb 17;13:958. Originally published 2024 Aug 23. [Version 4] doi: 10.12688/f1000research.153690.4 (PMC11842959; doi:10.12688/f1000research.153690.4)
Supplement: Peer review history

# Reviewer report: Reviewer response for version 4

**DOI:** 10.5256/f1000research.178081.r367034

**Published:** 2025-03-04

**Authors:** Matheus Koengkan

**Affiliations:** 1 University of Coimbra, Coimbra, Portugal

Accept in current form.

Is the work clearly and accurately presented and does it cite the current literature?

Partly

If applicable, is the statistical analysis and its interpretation appropriate?

Partly

Are all the source data underlying the results available to ensure full reproducibility?

Partly

Is the study design appropriate and is the work technically sound?

Partly

Are the conclusions drawn adequately supported by the results?

Partly

Are sufficient details of methods and analysis provided to allow replication by others?

Partly

Reviewer Expertise:

Macroeconomics, Latin America, Energy and Environmental Economics.

I confirm that I have read this submission and believe that I have an appropriate level of expertise to confirm that it is of an acceptable scientific standard.

# Reviewer report: Reviewer response for version 4

**DOI:** 10.5256/f1000research.178081.r367032

**Published:** 2025-02-28

**Authors:** Olimpia Neagu

**Affiliations:** 1 Universitatea de Vest, Arad, Romania

I confirm my APPROVED status for this paper.

Is the work clearly and accurately presented and does it cite the current literature?

Yes

If applicable, is the statistical analysis and its interpretation appropriate?

Yes

Are all the source data underlying the results available to ensure full reproducibility?

Yes

Is the study design appropriate and is the work technically sound?

Yes

Are the conclusions drawn adequately supported by the results?

Yes

Are sufficient details of methods and analysis provided to allow replication by others?

Yes

Reviewer Expertise:

Economic complexity, Economic modelling

I confirm that I have read this submission and believe that I have an appropriate level of expertise to confirm that it is of an acceptable scientific standard.

# Reviewer report: Reviewer response for version 4

**DOI:** 10.5256/f1000research.178081.r367031

**Published:** 2025-02-20

**Authors:** Abdul Jalil, Taqwa Syed, Dr. Muhammad Ibrar

**Affiliations:** 1 Department of Social Work, University of Peshawar (Ringgold ID: 54736), Peshawar, Khyber Pakhtunkhwa, Pakistan; 2 Department of Criminology, University of Peshawar (Ringgold ID: 54736), Peshawar, Khyber Pakhtunkhwa, Pakistan

**Summary of the Revised Manuscript:**

 I have carefully reviewed the revised version of the article "The Macroeconomic Determinants of Trade Openness in Latin American Countries: A Panel Data Analysis." The authors have made significant improvements to the manuscript in response to my previous feedback, as well as the suggestions provided by the other peer reviewers. Below is an assessment of the key revisions:

1. **Introduction:**
  - The authors have enhanced the coherence and clarity of the introduction, making it easier to follow. The study gap has been more explicitly defined, which helps the reader better understand the context and rationale of the research.
2. **Literature Review:**
  - The literature review has been substantially improved, adopting a more scholarly tone and clearer structure. The authors have separated the literature review from the theoretical foundations, ensuring better readability and organization.
3. **Methodology:**
  - The methodology section has been revised by merging subheadings, as per the reviewers' suggestions. This change contributes to a more streamlined and cohesive presentation of the research design and approach.
4. **Results Section:**
  - The results section has been rewritten to improve the clarity of the statistical tests and the presentation of findings. The authors have provided more in-depth discussion and interpretation of the results, which enhances the value of the analysis.
5. **Addressing Reviewer Observations:**
  - The authors have addressed the 50 observations raised in the original peer review, demonstrating a thorough and comprehensive revision process. These revisions have led to a more polished manuscript with greater academic rigor.

**Evaluation and Conclusion:**

 The revised manuscript now exhibits improved clarity, structure, and academic quality. The authors have successfully addressed the concerns raised during the peer review process, resulting in a more well-rounded and rigorous paper.

 Given the improvements made, I believe that the article is now suitable for indexing. I am therefore happy to approve the revised version of the manuscript.

**Approval Status:** Approved

Is the work clearly and accurately presented and does it cite the current literature?

Partly

If applicable, is the statistical analysis and its interpretation appropriate?

Yes

Are all the source data underlying the results available to ensure full reproducibility?

Partly

Is the study design appropriate and is the work technically sound?

Yes

Are the conclusions drawn adequately supported by the results?

Yes

Are sufficient details of methods and analysis provided to allow replication by others?

Partly

Reviewer Expertise:

Social Work and Community Development, Climate Change and Environmental Sustainability, Educational Strategies and Digital Literacy, Public Policy and International Relations

We confirm that we have read this submission and believe that we have an appropriate level of expertise to confirm that it is of an acceptable scientific standard.

# Reviewer report: Reviewer response for version 3

**DOI:** 10.5256/f1000research.174103.r362757

**Published:** 2025-01-29

**Authors:** Abdul Jalil, Taqwa Syed

**Affiliations:** 1 Department of Social Work, University of Peshawar (Ringgold ID: 54736), Peshawar, Khyber Pakhtunkhwa, Pakistan

**Summary of the Article**

 The article explores the impact of economic complexity, innovation, R&D, and industrial policies on trade openness in South America from 2000 to 2020. Using fixed-effects panel data analysis, the study identifies key challenges to trade openness, including limited diversification and insufficient innovation. The findings highlight the positive effects of R&D investment in countries like Chile and Brazil, while emphasizing the need for value-added industries and sustainable policies to enhance growth (Hausmann et al., 2007).

**Evaluation of the Article**

**Clarity and Accuracy of Presentation**

 The article is generally well-written and clear. However, the citation of current literature is limited. Studies published after 2020 should be included to provide a broader context, especially regarding post-pandemic economic trends.

**Appropriateness of Study Design and Technical Soundness**

 The study design and methodology are appropriate for the research objectives. The use of fixed-effects panel analysis effectively captures variations across countries and time. However, a more in-depth discussion of the robustness of the model would strengthen the technical soundness of the analysis.

**Details of Methods and Analysis**

 While the methods are described, there is insufficient detail for full replication. The data sources and variable definitions should be elaborated further. Additionally, the econometric model's assumptions and diagnostic checks need to be explicitly stated.

**Statistical Analysis and Interpretation**

 The statistical analysis is robust and the interpretation of findings is mostly sound. However, more nuanced discussion is needed for outliers like Venezuela, where political instability has likely influenced the results.

**Availability of Source Data**

 The study references reliable data sources but does not provide direct access to datasets or processing details. Publishing or linking these materials would improve transparency.

**Conclusions and Recommendations**

 The conclusions align with the results, but the recommendations could be more specific. For instance, suggesting targeted policies for fostering R&D, improving infrastructure, or diversifying exports in each country would enhance their practical relevance.

**Suggestions for Improvement**

1. Update the literature review with recent studies, especially those covering the post-2020 period.
2. Provide detailed descriptions of data processing, variable definitions, and econometric models to enhance reproducibility.
3. Make datasets and analysis scripts publicly available to improve transparency and future research opportunities.
4. Expand the discussion on country-specific findings, particularly for outliers like Venezuela, which may distort the regional analysis.
5. Strengthen the policy recommendations by tailoring them to individual country contexts with actionable strategies.

Is the work clearly and accurately presented and does it cite the current literature?

Partly

If applicable, is the statistical analysis and its interpretation appropriate?

Yes

Are all the source data underlying the results available to ensure full reproducibility?

Partly

Is the study design appropriate and is the work technically sound?

Yes

Are the conclusions drawn adequately supported by the results?

Yes

Are sufficient details of methods and analysis provided to allow replication by others?

Partly

Reviewer Expertise:

Social Work and Community Development, Climate Change and Environmental Sustainability, Educational Strategies and Digital Literacy, Public Policy and International Relations

We confirm that we have read this submission and believe that we have an appropriate level of expertise to confirm that it is of an acceptable scientific standard, however we have significant reservations, as outlined above.

## References

[1] : What you export matters. *Journal of Economic Growth* .2007;12(1) : 10.1007/s10887-006-9009-4 1-25 10.1007/s10887-006-9009-4

# Reviewer report: Reviewer response for version 3

**DOI:** 10.5256/f1000research.174103.r341858

**Published:** 2024-12-14

**Authors:** Matheus Koengkan

**Affiliations:** 1 University of Coimbra, Coimbra, Portugal

1. The abstract outlines the study's aims and provides a snapshot of the results. However, it lacks sufficient detail about the methodological approach and the numerical outcomes. Explicitly mention key quantitative findings, such as the percentage changes in trade openness associated with specific variables, to enhance its clarity and appeal.
2. The introduction provides a thorough context regarding trade liberalization in Latin America, but the central research problem could be articulated more succinctly. Clarify how the study addresses specific gaps in the literature and its unique contribution compared to prior studies.
3. The literature review is comprehensive but somewhat dense. Consider organizing it thematically, focusing on key determinants of trade openness (e.g., economic complexity, innovation) and their relevance to the Latin American context. This restructuring will improve readability and flow.
4. The methodology section is detailed but could better justify the choice of the fixed-effects model. Explain why this model was preferred over random effects or alternative econometric techniques in addressing the study's research questions.
5. The use of panel data is appropriate for this study, but the choice of the time period (2000–2020) requires further justification. Discuss why this specific timeframe was selected and how it influences the study's applicability to current trade policy challenges.
6. The explanation of the econometric models is technically sound, but it would benefit from more intuitive explanations for non-specialist readers. For instance, provide a brief overview of how the Generalized Least Squares (GLS) model addresses heteroscedasticity and autocorrelation.
7. The study's reliance on publicly available data is a strength, but the manuscript should clarify potential limitations related to data quality or coverage. For example, discuss any challenges in capturing informal trade flows, which are significant in some Latin American economies.
8. The descriptive statistics section provides useful insights, but the discussion of key trends is underdeveloped. Highlight notable patterns, such as variations in trade openness across countries, and their potential implications.
9. The results section identifies significant variables affecting trade openness but does not adequately explore country-specific dynamics. For instance, why do Chile and Brazil perform better in terms of economic complexity compared to other countries in the region?
10. The discussion section effectively connects findings to existing literature but could delve deeper into the implications of negative relationships, such as those between GDP and trade openness. How might these results inform regional trade policies or economic strategies?
11. The manuscript notes that the economic complexity index negatively affects trade openness but does not sufficiently explore why this counterintuitive relationship exists. Consider integrating qualitative insights or case studies to provide a more nuanced explanation.
12. While the focus on South America is justified, the manuscript could benefit from a brief comparison with other regions to contextualize the findings. For example, how do the determinants of trade openness in Latin America differ from those in Asia or Africa?
13. The manuscript mentions that certain countries exhibit declining trade openness due to a lack of diversification in export baskets. Expanding this analysis with specific examples, such as Venezuela's reliance on oil exports, would enhance the discussion.
14. The policy recommendations are relevant but somewhat general. Provide specific actions that countries could take, such as investing in high-tech export industries or improving logistics infrastructure, to enhance trade openness.
15. The tables and figures are informative but could be better integrated into the text. For instance, directly reference key values or trends from the descriptive statistics table when discussing results to improve coherence.
16. The regression results indicate that research and development expenditure positively impacts trade openness, but this finding is not sufficiently elaborated upon. Discuss how R&D investments could be incentivized in resource-dependent economies.
17. The manuscript uses the logistics performance index as an explanatory variable, which is innovative. However, it does not fully address why this index negatively correlates with trade openness. Explore possible reasons, such as inefficiencies in logistics systems or methodological issues.
18. The theoretical framework section is robust but could benefit from a conceptual diagram. Visualizing the relationships between economic complexity, innovation, and trade openness would make the study's theoretical underpinnings more accessible.
19. The discussion of trade openness trends could be enriched by considering external shocks, such as the global financial crisis or the COVID-19 pandemic, and their impacts on the study period.
20. The manuscript concludes by highlighting the importance of innovation and industrialization but does not adequately address the political and institutional barriers that might hinder these processes. Include a discussion on governance or institutional quality.
21. The use of econometric terminology is precise but may be challenging for readers unfamiliar with the field. Consider adding a glossary or briefly defining terms like "heteroscedasticity" and "autocorrelation" in the methodology section.
22. The manuscript references several influential studies but could incorporate more recent research on trade openness and economic complexity, particularly studies published after 2020.
23. While the conclusions are aligned with the findings, they could be more forward-looking. Suggest avenues for future research, such as examining the role of digital trade or regional trade agreements in shaping trade openness.
24. The ethical considerations section is clear but could explicitly address any potential biases in data collection or analysis. This would strengthen the study's credibility.
25. The language is generally clear, but there are instances of verbosity. Streamline sentences for clarity and impact, particularly in the introduction and literature review.
26. The manuscript does not address the role of human capital in trade openness, despite its potential relevance. Discuss whether factors like education levels or workforce skills influence the results.
27. The choice of explanatory variables is appropriate, but the manuscript could discuss alternative variables that might also affect trade openness, such as exchange rate volatility or foreign direct investment.
28. The manuscript identifies a positive relationship between exports of high-tech products and trade openness but does not explore the underlying mechanisms. For example, how do high-tech exports foster trade liberalization?
29. The discussion section mentions diminishing returns to physical capital but does not elaborate on how this affects long-term trade openness. Expanding this point would provide a more comprehensive analysis.
30. The study notes that some countries exhibit lower trade openness due to political shifts but does not provide detailed examples. Include specific instances of how political changes have influenced trade policies in the region.
31. The manuscript cites several classical economic theories but does not explicitly link them to the findings. For instance, how does the Solow growth model relate to the observed impacts of innovation and R&D?
32. The results highlight the role of imports in promoting trade openness, but the manuscript does not discuss the quality or composition of imports. Are certain types of imports (e.g., capital goods) more influential?
33. The study's focus on South America is justified, but the manuscript could explore whether its findings are applicable to other regions with similar economic structures.
34. The manuscript notes that trade openness has declined in some countries due to economic crises but does not address how these crises could be mitigated. Provide policy recommendations to address economic vulnerability.
35. The regression results are robust, but the manuscript could include additional diagnostics, such as tests for multicollinearity, to strengthen the validity of the findings.
36. The study's emphasis on innovation is commendable, but the manuscript could discuss the role of intellectual property rights or patent systems in fostering innovation.
37. The manuscript does not explore the environmental implications of trade openness, despite its relevance to sustainable development. Include a brief discussion on how trade policies could align with environmental goals.
38. The conclusion mentions that trade openness fosters economic growth but does not discuss the potential trade-offs, such as income inequality or labor market disruptions.
39. The manuscript could benefit from a stronger narrative thread connecting the introduction, methodology, results, and discussion. Ensure that each section builds logically on the previous one.
40. The literature review cites numerous studies but could include more critical evaluations of their methodologies or findings to highlight the study's unique contributions.
41. The manuscript does not address the potential role of digital technologies, such as e-commerce platforms, in influencing trade openness. Discuss their relevance, especially in the post-pandemic era.
42. The policy recommendations are insightful but could be supplemented with examples of successful trade policies from other regions.
43. The manuscript could include a sensitivity analysis to test the robustness of the regression results under different model specifications.
44. The findings suggest that countries with higher economic complexity exhibit greater trade openness, but the manuscript does not discuss how complexity can be enhanced in less developed economies.
45. The study focuses on macroeconomic variables but does not consider microeconomic factors, such as firm-level competitiveness or supply chain efficiency. Discuss their potential relevance.
46. The manuscript uses the Breusch-Pagan and Wooldridge tests effectively but does not explain their implications for model selection. Provide more detail on why these tests were necessary.
47. The conclusion could be enhanced by discussing the broader implications of the findings for global trade policies, particularly in the context of South-South cooperation.
48. The manuscript identifies significant country-specific differences in trade openness but does not explore potential regional collaborations to address shared challenges.
49. The study's emphasis on research and development is valuable, but the manuscript could discuss the role of public-private partnerships in fostering innovation.
50. The manuscript could benefit from professional proofreading to address minor grammatical errors and improve readability.

Is the work clearly and accurately presented and does it cite the current literature?

Partly

If applicable, is the statistical analysis and its interpretation appropriate?

Partly

Are all the source data underlying the results available to ensure full reproducibility?

Partly

Is the study design appropriate and is the work technically sound?

Partly

Are the conclusions drawn adequately supported by the results?

Partly

Are sufficient details of methods and analysis provided to allow replication by others?

Partly

Reviewer Expertise:

Macroeconomics, Latin America, Energy and Environmental Economics.

I confirm that I have read this submission and believe that I have an appropriate level of expertise to state that I do not consider it to be of an acceptable scientific standard, for reasons outlined above.

# Reviewer report: Reviewer response for version 1

**DOI:** 10.5256/f1000research.168614.r323042

**Published:** 2024-10-18

**Authors:** Kingsley Appiah

**Affiliations:** 1 Kumasi Technical University, Kumasi, Ashanti Region, Ghana

**Introduction**

 1.The statement at the introduction section such as ***‘Trade openness in countries around the world has shown significant relevance in addressing one of the greatest global crises, where the acute and real problem of global food security, coupled with rising inflation, demonstrates that trade policies not only ensure adequate access to food but also compel a consideration of the link with economic complexity, which in turn requires research and development and the creation of an innovation climate that impacts economic growth (Bardi & Hfaiedh, 2021; Fusco et al., 2020; Gallego & Zofío, 2018; Tabash et al., 2022)’***.

 The statement is too long which ends up distorting what the researchers are trying to communicate. I suggest that the authors make it brief.

 2. I suggest that authors improve on the flow of the write-up and how the various paragraphs under the introduction section are connected to make much more sense. There is a lack of coherence in the introductory section which makes it difficult to understand what the authors are trying to send around.

 3. In terms of the study gap identified, authors should bring into perspective what has already been done in the study areas and what this particular study seeks to address as a gap.

**Literature review**

 4. The same can be said about the literature section of the study. The authors failed to connect the various scholars' work to make the section more coherent and interesting. Although authors made use of current research work by scholars the literature strand depicts a write-up of other researchers' work put into paragraphs. I suggest that the authors improve on the literature section of the study.

 5. I suggest that authors classify the literature section into empirical and theoretical study

 6. I suggest authors move the discussion of the variables under study from the theoretical basis section to theoretical study under a subheading named conceptual terms. At the same time, 3.1 and 3.2 are under the subheading ‘study theories’.

**Methods**

 7. I suggest authors merge the following subheadings and put them under these subheadings title:

 Merge 4.1.1 & 4.1.2 as 4.1 Research design

 Merge 4.1.3, 4.1.4 & 4.1.5 as 4.2 Population and sampling

 Merge 4.1.6 & 4.1.7 as 4.3 Data collection technique & procedure

**Results**

 8. Since the authors’ analysis is based on a panel dataset, I expected them to perform certain fundamental or preliminary tests, such as the cross-sectional dependency test, unit root test, and cointegration test. I suggest that the authors perform these basic tests and discuss them in the results section while describing them in the methods section.

 9. Proofreading the work would help.

Is the work clearly and accurately presented and does it cite the current literature?

Partly

If applicable, is the statistical analysis and its interpretation appropriate?

Partly

Are all the source data underlying the results available to ensure full reproducibility?

Partly

Is the study design appropriate and is the work technically sound?

Partly

Are the conclusions drawn adequately supported by the results?

Partly

Are sufficient details of methods and analysis provided to allow replication by others?

No

Reviewer Expertise:

Environmental management, Sustainability management, Panel data modelling

I confirm that I have read this submission and believe that I have an appropriate level of expertise to state that I do not consider it to be of an acceptable scientific standard, for reasons outlined above.

# Response

**Published:** 2025-02-04

**Authors:** Rogger Orlando Morán Santamaría

**Affiliations:** Universidad Cesar Vallejo, Trujillo, La Libertad, Peru

**Kingsley Appiah**

 Kumasi Technical University, Kumasi, Ashanti Region, Ghana

 Please accept our best regards from the authors of the article.

 We appreciate your comments and know that they improve the quality of our finalised research.

 I am writing to inform you that the report has been uploaded with the suggested changes and that we can continue with the process of publishing the article to the highest standards of quality.

 We hope we have met your requirements.

 We look forward to hearing from you.

 Thank you very much.

# Reviewer report: Reviewer response for version 1

**DOI:** 10.5256/f1000research.168614.r323039

**Published:** 2024-09-26

**Authors:** Olimpia Neagu

**Affiliations:** 1 Universitatea de Vest, Arad, Romania

**General comments:**

 The paper is appropriately conceived and structured.

 Introduction section is well developed.

 Literature review cites the current literature on the macroeconomic determinants of trade openness.

 Variables selection is justified and the methodology design is explained

 Conclusions are supported by the paper’s results.

**Recommendations:**

 I suggest to rename the Section 4. Methods in: 4. Methodology and data

 Title 4.2 is in Spanish, please correct it.

Is the work clearly and accurately presented and does it cite the current literature?

Yes

If applicable, is the statistical analysis and its interpretation appropriate?

Yes

Are all the source data underlying the results available to ensure full reproducibility?

Yes

Is the study design appropriate and is the work technically sound?

Yes

Are the conclusions drawn adequately supported by the results?

Yes

Are sufficient details of methods and analysis provided to allow replication by others?

Yes

Reviewer Expertise:

Economic complexity, Economic modelling

I confirm that I have read this submission and believe that I have an appropriate level of expertise to confirm that it is of an acceptable scientific standard.
